# Urbanization gradient, diet, and gut microbiota in Sub-Saharan Africa: a systematic review

**DOI:** 10.3389/frmbi.2023.1208166

**Published:** 2023-09-12

**Authors:** Linda Simon Paulo, George Msema Bwire, K. Klipstein-Grobusch, Appolinary Kamuhabwa, Gideon Kwesigabo, Pilly Chillo, Folkert W. Asselbergs, Virissa C. Lenters

**Affiliations:** ^1^ Julius Global Health, Julius Center for Health Sciences and Primary Care, University Medical Center Utrecht, Utrecht University, Utrecht, Netherlands; ^2^ Cardiac Center of Excellence, Muhimbili University of Health and Allied Sciences, Dar Es Salaam, Tanzania; ^3^ Department of Pharmaceutical Microbiology, School of Pharmacy, Muhimbili University of Health and Allied Sciences, Dar Es Salaam, Tanzania; ^4^ Division of Epidemiology and Biostatistics, School of Public Health, Faculty of Health Sciences, University of the Witwatersrand, Johannesburg, South Africa; ^5^ Institute for Tropical Medicine, University of Tübingen, Tübingen, Germany; ^6^ Department of Clinical Pharmacy and Pharmacology, School of Pharmacy, Muhimbili University of Health and Allied Sciences, Dar Es Salaam, Tanzania; ^7^ Department of Biostatistics and Epidemiology, School of Public Health and Social Sciences, Muhimbili University of Health and Allied Sciences, Dar Es Salaam, Tanzania; ^8^ Department of Cardiology, Jakaya Kikwete Cardiac Institute, Muhimbili, Dar Es Salaam, Tanzania; ^9^ Amsterdam University Medical Centers, Department of Cardiology, University of Amsterdam, Amsterdam, Netherlands; ^10^ Health Data Research United Kingdom (UK) and Institute of Health Informatics, University College London, London, United Kingdom; ^11^ Department of Environment and Health, Amsterdam Institute for Life and Environment, Vrije Universiteit Amsterdam, Amsterdam, Netherlands

**Keywords:** gut microbiota, human microbiome, urbanization, Sub-Saharan Africa, diet, nutrition transition, intestinal parasites

## Abstract

**Introduction:**

As Sub-Saharan Africa (SSA) undergoes rapid urbanization changes in diet and lifestyle have contributed to a rise in non-communicable diseases (NCDs) across the region. Changes in gut microbiota which play an important role in human health may be an underlying driving factor. While evidence suggests that the gut microbiota differs between the extreme levels of economic development (least vs highly developed), it is not well-established which factors along the urbanization gradient are most influential, especially for SSA. This systematic review analyzed published articles from SSA countries that examined the differences in the composition and diversity of gut microbiota along the urbanization gradient. The findings of this review have important implications for understanding the impact of urbanization on human health in the SSA.

**Methods:**

Peer-reviewed articles that examined the link between the urbanization gradient, dietary patterns, and gut microbiota using culture-independent techniques were included in the review.

**Results:**

A total of 3,265 studies were identified and screened. Eighty-nine (89) studies underwent full-text review, and 23 studies were extracted and included for final analysis. Among these studies, it was observed that hunter-gatherers had high alpha diversity (within-person variation) and beta diversity (between-person variation) in their gut microbiota compared to rural and urban residents in SSA. However, there were inconsistent differences between rural and urban at the individual taxa levels, potentially due to limited statistical power and large variability in the study techniques and designs. Similarly, there were no clear differences in the relative abundance of genera across the urbanization gradient. Additionally, both diet and intestinal parasites were associated with the composition and diversity of the gut microbiota.

**Conclusion:**

The review revealed there are variations in both alpha and beta diversity of the gut microbiota across the urbanization gradient with a higher diversity observed in rural areas. However, we did not observe significant differences in the relative abundance at phyla or genus levels consistently across the urbanization gradient. Moreover, our findings suggest that the mode of subsistence, diet, and intestinal parasites play a role in shaping the composition and diversity of the gut microbiota in SSA.

**Systematic review registration:**

https://www.crd.york.ac.uk/prospero/display_record.php?ID=CRD42021251006, identifier CRD42021251006.

## Introduction

Frequent contact with the natural environment including soil and vegetation is postulated to enrich the diversity and composition of the gut microbiota (Mancabelli et al., 2017; Fragiadakis et al., 2019). High interaction with the natural environment has remained a major characteristic of rural/traditional communities giving rise to the term “rural microbiome” (Du et al., 2021). The rural microbiome is hypothesized to enhance health and protect against chronic illnesses (Blaser, 2017; Zuo et al., 2018). The number of rural/traditional communities continues to decline globally due to rapid urbanization, population growth, and technological advances (Ilbery, 2014; Li et al., 2019). The SSA is among the remaining geographical regions with transitioning communities at different levels of interaction with the natural environment such as; pre-agriculture, agro-pastoralists, nomadic pastoralists, and communities in rural and urban areas (Njoh, 2003; Kassouri and Okunlola, 2022). Overall, SSA is undergoing rapid urbanization, and population growth and sees an increasing burden of NCDs (Popkin, 1998; Steyn and McHiza, 2014). As such, the role of altered microbiomes in driving the increase in NCDs deserves attention.

Urbanization and modernization have several features that are linked to the changes in the composition and diversity of the gut microbiota (Yatsunenko et al., 2012; Morton et al., 2015; Mancabelli et al., 2017; Jha et al., 2018). For example, the frequency of using antimicrobials, exposure to cleaning chemicals, less interaction with the natural environment, population density, and excessive cleaning increases as one moves from traditional/hunter-gatherers to urbanized communities (Yatsunenko et al., 2012; Jha et al., 2018; Fragiadakis et al., 2019; McCall et al., 2020; Gacesa et al., 2022; Rosas-Plaza et al., 2022). The difference between urban and rural living environments is also evident in dietary patterns as urban residents more often eat processed foods with lower contents of fibers compared to traditional or other rural communities (David et al., 2014; Cockx et al., 2018). They also have access to a wider variety of foods and dishes compared to their counterparts (Cockx et al., 2018).

The gut microbiota plays a complex key role in human metabolism through different biochemical activities (Bäckhed et al., 2012; Doumatey et al., 2020). While no single optimal healthy composition and diversity of the gut microbiota in humans are known, there is increasing evidence for various sub-optimal microbiota compositions. And several probiotics, prebiotics, and post-biotic supplements to balance the gut microbiota are already in use (Fan and Pedersen, 2021; Oniszczuk et al., 2021; Zhang et al., 2021). Different healthy populations around the world have been found to possess unique core gut microbiota compositions and diversity (Sekirov et al., 2010; King et al., 2019; Lu et al., 2021). Similar to diets, there is no singular best gut microbiota, but it is essential to maintain important functions and resilience.

Evidence exists on the differences in the composition and diversity of the gut microbiota between highly and least industrialized communities (Gupta et al., 2017; Mancabelli et al., 2017). Generally, residing in industrialized communities is linked with less diverse gut microbiota and altered composition at different taxa levels (Gupta et al., 2017; Mancabelli et al., 2017). However, this may not reflect the situation in transitioning communities for example in SSA where there is a co-existence of traditional hunter-gatherers, pastoralists, agro-pastoralists, and rural and urban communities (Morton et al., 2015; Lokmer et al., 2020; Even et al., 2021). Single studies done in other geographical regions (China, India, South America, and Europe) in rural/urban communities have shown mixed results (Tyakht et al., 2014; Ramadass et al., 2017; Das et al., 2018; Li et al., 2018; McCall et al., 2020; Cui et al., 2021). Hence synthesized evidence on the gut microbiota across the urbanization gradient is still needed to expand the knowledge of the drivers of NCDs.

This systematic review analyzed published articles from SSA countries that examined the differences in the composition and diversity of gut microbiota of healthy people along the urbanization gradient. It aims at examining the differences in the gut microbiota and determining the potential explanatory factors that influence the gut microbiota of the residents in Sub-Saharan African countries.

## Methods

### Identification and selection of articles

We conducted a PROSPERO-registered systematic review (CRD42021251006) of peer-reviewed articles that examined the link between the urbanization gradient, dietary patterns, and gut microbiota. We searched PubMed/Medline, Scopus, Web of Science, and Embase databases. Three main concepts were used in search studies: gut microbiota, urbanization, and Sub-Saharan African countries (refer to **Supplementary Material Appendix 1** for the search strings). Studies were included if they met the inclusion criteria: observational and interventional studies, restricting to control groups of case-control studies or baseline data of randomized controlled trials; published in English from 2000 to 2022; and used a culture-independent identification (i.e., next-generation sequencing) of gut microbiota and studied adult populations. Searches were run up to 1 March 2022. Two independent reviewers (LSP and GMB) performed title and abstract screening using Rayyan (Ouzzani et al., 2016). Quality assessment and characteristics of the included studies were extracted, and a full-text review was conducted using a structured online form (https://app.covidence.org/).

### Assessment of quality and bias of included studies

We used a modified Newcastle–Ottawa Scale (NOS) to assess the quality of cross-sectional and case-control studies that consider the selection of study participants, comparability, and outcome features related to the microbiome assessment (**Table 1** and **Supplementary Material Appendix 2**), as previously used (van den Munckhof et al., 2018; Gong et al., 2021). No intervention studies met the inclusion criteria. As no study was critically biased, none was excluded from the review.

**Table 1:** A summary of included studies, sample characteristics, and methodologies of the included studies

Author	Subsistence gradient	Country	Sample size	Age -mean(range)	Sequenced region	DNA extraction	Sequencing platform	Analysis pipeline	Functional prediction	Quality score
Smits et al., 2017	Hunter-gatherers	Tanzania	58	adults	16S rRNA (V1-V5)	Powersoil-htp extraction kits	Illumina, MISEQ	QIIME	HUMAnN2	Fair
Schnorr et al., 2013		Tanzania	27	32 (8-70)	16S rRDA(V4)	QIAamp DNA Stool Mini Kit	454 Pyrosequencing	QIIME	GC–MS determination of SCFAs in faecalsamples	Fair
Gomez et al., 2016	Hunter- gatherers/Agropastoral/subsistence farmers	Central Africa	57	adults	16S rRNA (V1-V3)	MoBio Ultraclean Soil Kit	454 pyrosequencing	QIIME	PiCRUST	Fair
Angelakis et al., 2019	Hunter-gatherers/ semi- urban/urban	Senegal	177	>18	16S rRNA	NucleoSpin Tissue Mini Kit(Macherey Nagel, Hoerdt, France)	Illumina, MISEQ	QIIME	PiCRUST	Fair
Morton et al., 2015	Hunter-gatherers/Rural/ Agropastoral/subsistence farmers	Cameroon	64	50(26-78)	16S rRNA (V5-V6)	MOBIO PowerFecal DNAIsolation Kit	Illumina, MISEQ	QIIME	PiCRUST	Fair
Rubel et al., 2020		Cameroon	575	adults	Shotgun metagenomics and16S rRNA (V4)	PSP Spin Stool DNA Plus Kit	Illumina, MISEQ	QIIME2	shotgun metagenomics	Fair
Hansen et al., 2019		Tanzania &Botswana	114	44.5-Tanzania;40.9- Botswana (18-92)	16SrRNA (V1, V2)	MOBIO PowerSoil DNA Isolation Kit & PSP Spin StoolDNA Plus Kit	Illumina, MISEQ	QIIME	PiCRUST	Fair
Chen et al., 2021	Rural	Tanzania	32	29.27(23-45)	16S rRNA	PureLink™ Microbiome DNAPurification Kit	Illumina, MISEQ	QIIME2	PiCRUST	Fair
Dugas et al., 2018		Ghana	50	adults	16S rRNA (V4)	DNeasy PowerSoil HTP 96 Kit	Illumina HiSeq. 2500	QIIME	Piphillin	Fair
Ellis et al., 2013		Uganda	15	30-45	16S rRNA (V4 - V5)	QIAsymphony automatedextraction platform + chemical lysis	454 pyrosequencing	QIIME	not done	Fair
Tang et al., 2019		DRC	117	16-35	16S rRNA (V3-V4)	QIAamp PowerFecal DNA kit	Illumina, MISEQ	SINA (1.3.0-r23838)	not done	Fair
Rosa et al., 2018	Rural/semi-urban	Liberia	98	26	16S rRNA (V1-V3)	not found	Illumina MISEQ & 454pyrosequencing	Mothur	shotgun metagenomics	Good
Even et al., 2021	Rural/semi-urban/urban	Cameroon	134	38(18-64)	16S rRNA	MOBIO PowerFecal DNAIsolation Kit	Illumina, MISEQ	QIIME2	not done	Fair
Lokmer et al., 2020		Cameroon	147	18-65	16S rRNA (V4)	MOBIO PowerFecal DNAisolation kit	Illumina, MISEQ	Mothur	not done	Fair
Parbie et al., 2021	Rural/urban	Ghana	55	33-51	16S rRNA (V3-V4)	not found	Illumina, MISEQ	QIIME2	not done	Fair
Katsidzira et al., 2019		Zimbabwe	10	urban - 61.6;rural-65.3	16S rRNA	Qiagen DNA extraction kit	HITChip	Agilent FeatureExtraction software	not done	Good
Ocvirk et al., 2020		South Africa	21	53.3(40-65)	16S rRNA (V4)	Qiagen DNA stool Mini Kit	Illumina, MISEQ	Mothur	not done	Fair
Oduaran et al., 2020		South Africa	170	Urban-54.1;Rural-55.5 (43-72)	16S rRNA (V3-V4)	Qiagen DNA extraction kit	Illumina, MISEQ	QIIME2	not done	Fair
Ayeni et al., 2018		Nigeria	48	3-75 years	16S rRNA (V3-V4)	Bead beating & extractionusing QIAamp DNA Stool Mini Kit	Illumina, MISEQ	PANDAseq and QIIME	not done	Fair
Lebba et al., 2016		Cote d'Ivoire	20	21.7(1-74)	16S rRNA (V6-V8)	QIAmp Stool Mini Kit	gel electrophoresis	PATRIC	not done	Fair
Afolayan et al., 2019	Rural/urban/Agropastoral/subsistence	Nigeria	50	urban - 29;rural -19 (2-70)	16S rRNA (V4)	MagnaPure LC DNA IsolationKit III	Illumina, MISEQ	QIIME	PiCRUST	Fair
Afolayan et al., 2020	Urban	Nigeria	22	68.67	16S rRNA(V4)	MagnaPure LC DNA IsolationKit III	Illumina, MISEQ	QIIME2	PiCRUST	Fair
Doumatey et al., 2020*		Nigeria	291	Adults	16S rRNA(V4)	MoBioPowerMagRMicrobiome kit	Illumina, MISEQ	UPARSE	Piphillin	Good

* - case control study.

## Results

### General characteristics of included studies

**Figure 1:**
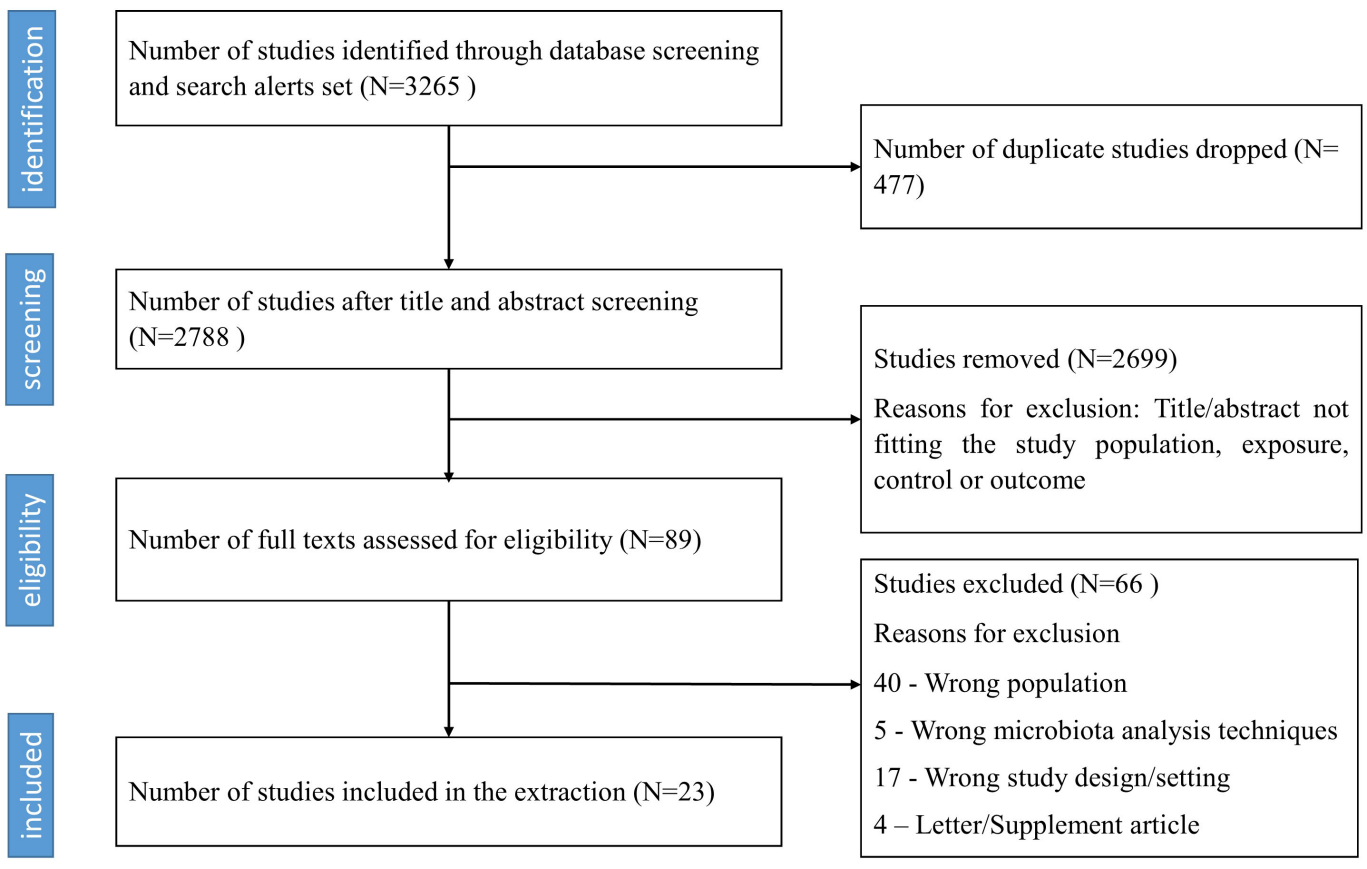
PRISMA-based flow chart of identified and included studies.

A total of 3,265 studies were identified, 89 studies were eligible for full-text review, and 23 studies met the inclusion criteria as shown in **Figure 1**.

The characteristics of the studies are summarized in **Supplementary Material Table 1**. The studies included 22 cross-sectional studies (Ellis et al., 2013; Schnorr et al., 2014; Morton et al., 2015; Gomez et al., 2016; Iebba et al., 2016; Smits et al., 2017; Ayeni et al., 2018; Dugas et al., 2018; Rosa et al., 2018; Afolayan et al., 2019; Angelakis et al., 2019; Hansen et al., 2019; Katsidzira et al., 2019; Tang et al., 2019; Afolayan et al., 2020; Lokmer et al., 2020; Ocvirk et al., 2020; Oduaran et al., 2020; Rubel et al., 2020; Chen et al., 2021; Even et al., 2021; Parbie et al., 2021) and 1 case-control study (Doumatey et al., 2020), representing a total of 2,372 individuals. For the case-control study, we considered only the control group that assessed gut microbiota in the adult population with and without diabetes (Doumatey et al., 2020). Most studies (Morton et al., 2015) originated from countries in West Africa (n=13 studies; (Nigeria (Ayeni et al., 2018; Afolayan et al., 2019; Afolayan et al., 2020; Doumatey et al., 2020), Ghana (Dugas et al., 2018; Parbie et al., 2021), Cameroon (Morton et al., 2015; Lokmer et al., 2020; Rubel et al., 2020; Even et al., 2021), Cote d’Ivoire (Iebba et al., 2016), Senegal (Angelakis et al., 2019), Liberia (Rosa et al., 2018)) followed by East Africa (n=5 studies; (Tanzania (Schnorr et al., 2014; Smits et al., 2017; Hansen et al., 2019; Chen et al., 2021) and Uganda (Ellis et al., 2013)), Southern Africa (n=3 studies; (South Africa (Ocvirk et al., 2020; Oduaran et al., 2020) and Zimbabwe (Katsidzira et al., 2019)) and Central Africa (n=2 studies; (Central Africa Republic (Gomez et al., 2016) and DRC (Tang et al., 2019)). Seven studies included participants practicing hunting-gathering, while others combined rural, subsistence farmers, or urban-based populations. The age of adult participants ranged from 18-78 years although it was not defined in four studies (Gomez et al., 2016; Smits et al., 2017; Dugas et al., 2018; Rubel et al., 2020).

Of the 23 included studies, three were classified as having good quality (Rosa et al., 2018; Katsidzira et al., 2019; Doumatey et al., 2020) while the rest were ranked as fair quality. Studies ranked as good described the sampling strategies and justified the sample size used (Rosa et al., 2018; Katsidzira et al., 2019; Doumatey et al., 2020). These features were missing in other studies that scored fair. All studies scored high in the use and description of the measurement tools, ascertainment of outcomes (i.e., gut microbiota), and measurement of association between the exposure and outcome factors. However, the heterogeneity in comparison groups and microbiota measures precluded doing a meta-analysis.

### Human settlements: definitions of the rural-urban gradient and urbanization

Several approaches have been used to define urbanization and quantify the urban-rural gradient considering different features such as population density, administrative status, land cover, and socioeconomic activities (Urbanization - Overview | US EPA; Gibbs, 1966; Li and Lu, 2021). In examining the relationship between the different environmental factors and gut microbiota scientists have used the words urban, rural, and subsistence patterns in isolation or as an index measure by combining different attributes (Obregon-Tito et al., 2015; Mancabelli et al., 2017).

In the included studies (refer to **Table 1**), urban was defined as highly urbanized (Oduaran et al., 2020) or based on the consumption of industrially processed foods or a Westernized lifestyle (Ayeni et al., 2018; Afolayan et al., 2019; Afolayan et al., 2020). Rural was classified in terms of subsistence patterns such as transitioning rural (Oduaran et al., 2020), cattle keepers for pastoralists and nomadic pastoralists or agro-pastoralists described as cattle keepers who cultivate crops (Afolayan et al., 2019; Rubel et al., 2020), agriculturalists with western-like subsistence pattern (Gomez et al., 2016), agrarian community (Ayeni et al., 2018), and rural farming and fishing communities (Morton et al., 2015). Hunter-gatherers were referred to as small-scale farmers who hunt and gather food (Rubel et al., 2020) or simply hunter-gatherers (Morton et al., 2015; Gomez et al., 2016; Ayeni et al., 2018). Lokmer et al. created an index to assess the urbanization gradient based on the availability of electricity, use of tap water, housing floor, in-house animal rearing, use of antibiotics, and the level of education (Lokmer et al., 2020). Other included studies used urban or rural without further characterization.

## Microbiome analysis: alpha and beta diversity, differential abundance, and functional analysis

### DNA extraction

The procedures and kits used to extract DNA from stool samples were reported in 21 studies but were not explicitly described in 2 studies (Rosa et al., 2018; Parbie et al., 2021). The most used extraction kit was from Qiagen (8 studies) followed by the MOBIO isolation kit (6 studies). Approaches used for DNA lysis varied between studies as some used mechanical bead beating (Dugas et al., 2018; Hansen et al., 2019; Katsidzira et al., 2019; Rubel et al., 2020) while others used both mechanical and chemical lysis (Tang et al., 2019) or chemical lysis alone (Iebba et al., 2016). It was not clearly stated in the rest of the studies whether mechanical/chemical lysis was applied in addition to the extraction protocol used.

### Sequencing

All studies sequenced the 16S rRNA gene using at least one of the variable regions (V1-V9), although some studies did not report the exact region that was sequenced (Angelakis et al., 2019; Katsidzira et al., 2019; Chen et al., 2021; Even et al., 2021). V4 was the most sequenced region reported in 13 studies. In addition to 16S rRNA, Rubel et al. used shotgun metagenomics (Rubel et al., 2020). Sequencing the entire 16S rRNA gene is associated with a higher bacteria diversity although a higher yield is also seen with the V4 region compared to other regions. Most studies used Illumina, Miseq as a sequencing platform and QIIME and QIIME2 for sequence analysis except a few (GS FLX Titanium platform (Ellis et al., 2013), 454 pyrosequencing (Schnorr et al., 2014; Gomez et al., 2016; Rosa et al., 2018), HITChip (Katsidzira et al., 2019) and gel electrophoresis (Iebba et al., 2016).

### Gut microbiota quantification

The total reads ranged from 50,000 – 51,655,653 while the average reads per sample ranged from 6,000 - 93,171.06. Overall, the reporting of the reads varied and was not reported in half of the studies identified.

### Diversity measures and functional analysis

Various methods were used to assess the diversity of the gut microbiota. Beta diversity (variation between samples, reflecting (dis)similarity in community composition of groups) was assessed in 17 studies, and measures including Bray-Curtis, Unweighted UniFrac distance, and Weighted UniFrac distance were reported in 17 studies. Alpha diversity (within-sample diversity) was assessed in 20 studies and measures including Shannon, Chao1, Simpson, and OTU richness. Predictive functional profiling was most frequently performed using “Phylogenetic Investigation of Communities by Reconstruction of Unobserved States” (PiCRUST) in 7 studies followed by shotgun metagenomics (Rosa et al., 2018; Rubel et al., 2020), Piphilin (Dugas et al., 2018; Doumatey et al., 2020) and HUMAnN2 (Smits et al., 2017). Other approaches include the use of gas-chromatography–mass spectrometry (GC–MS) to determine short-chain fatty acids (SCFAs) in fecal samples (Schnorr et al., 2014). Ten studies did not perform functional analysis.

#### Alpha and beta diversity

Three studies reported alpha diversity across an urbanization gradient whereby one study compared urban and rural (Oduaran et al., 2020), the second compared urban, semi-urban, and rural (Lokmer et al., 2020), and the third one compared hunter-gatherers versus rural communities-pastoralists and subsistence farmers (Rubel et al., 2020). Lokmer et al. showed an overall decrease in alpha diversity along the urbanization gradient (Lokmer et al., 2020). These findings were echoed by Oduaran et al. where alpha diversity was higher in rural compared to urban communities (Oduaran et al., 2020). The hunter-gatherers in Rubel et al. (2020) showed higher alpha diversity compared to other communities while the pastoralists had the lowest alpha diversity measures (Rubel et al., 2020). Overall, the alpha diversity was higher in hunter-gatherers/rural compared to urban and lower in pastoralists compared to other rural communities.

Beta diversity was presented in five studies (Gomez et al., 2016; Ayeni et al., 2018; Afolayan et al., 2019; Angelakis et al., 2019; Oduaran et al., 2020). Two studies compared hunter-gatherers versus urban communities (Gomez et al., 2016; Angelakis et al., 2019), and both showed a significant difference in beta diversity revealing greater diversity in hunter-gatherer populations. Differences in the diversity measures between urban and rural were also shown in Oduaran et al. (p=0.001 for Bray-Curtis dissimilarity measure) and Ayeni et al. (p<0.05 for Unweighted and Weighted UNIFRAC distances) with higher diversity in rural communities (Ayeni et al., 2018; Oduaran et al., 2020). This is similarly reported in Afolayan et al. where the pastoralists had a more diverse gut microbiome compared to the urban residents’ (Afolayan et al., 2019).

### Differential abundance analysis at different taxa levels along the urbanization gradient

#### Phylum and family level

**Figure 2:**
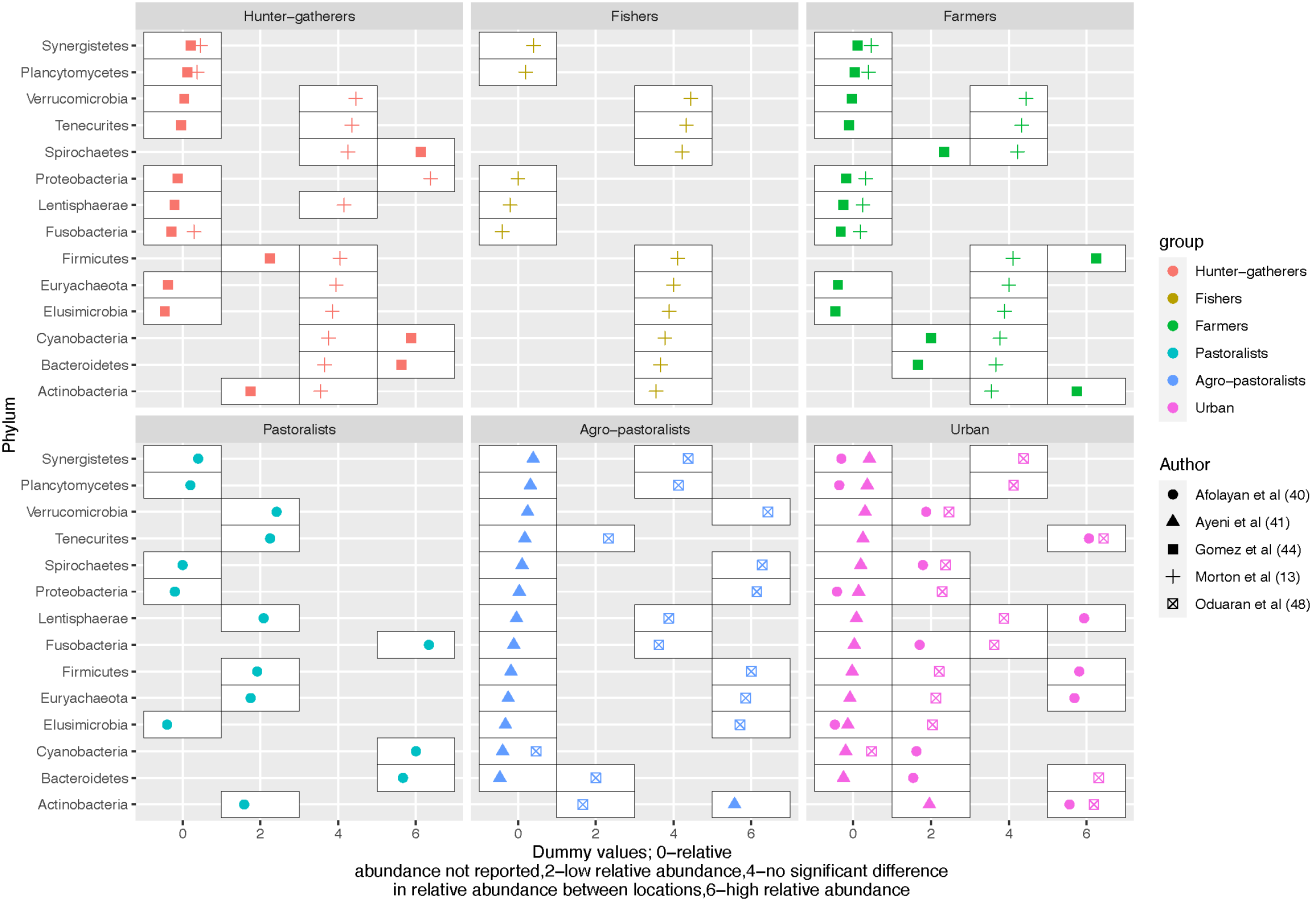
An overview of the differences in relative abundance at phylum level across the urbanization gradient.

Reporting of the composition of gut microbiota varied between studies. Therefore, complete mapping of the gut microbiota across the hunter-gatherers, rural and urban gradients is limited. Out of the included studies, 5 (4 studies included rural and 3 in hunter-gatherers’ settings) reported 6 phyla in common (higher taxonomic level); Firmicutes, Bacteroidetes, Cyanobacteria, Spirochaetes, Actinobacteria and Proteobacteria (Schnorr et al., 2014; Gomez et al., 2016; Afolayan et al., 2019; Tang et al., 2019; Chen et al., 2021). Firmicutes and Bacteroidetes were the most abundant phyla in both settings. The range for Firmicutes in rural vs hunter-gatherers areas was (46.9-77.63%) vs (49.5-72%) respectively; the range for Bacteroidetes also among rural and hunter-gatherers areas was (13.8-33%) vs (17-44.4%) respectively (Schnorr et al., 2014; Gomez et al., 2016; Afolayan et al., 2019; Tang et al., 2019; Chen et al., 2021). This was followed by Proteobacteria which was higher in rural settings compared to hunter-gatherers, with a range of (5.5 -17.73%) in rural vs (6-8.49%) in hunter-gatherers. The presence of Actinobacteria was relatively low in both rural (1.27-1.79%) and hunter-gatherer populations (less than 0.54%). Spirochaetes were reported in all 3 studies focusing on hunter-gatherer populations, but their abundance was relatively low. In contrast, only 50% of the studies done in rural areas reported the presence of Spirochaetes, with their abundance ranging from low to high. Cyanobacteria were the least commonly reported in 1 study in each setting (0.29% rural) vs (0.7% Hunter-gatherers). Most phyla did not differ between hunter-gatherers and rural in Morton et al. except for Proteobacteria where the relative abundance was higher among hunter-gatherers. The details of the direction of the relative abundance at the phyla level reported across the gradient are shown in **Figure 2**.

Eight studies reported gut microbiota at the family level (Dugas et al., 2018; Afolayan et al., 2019; Hansen et al., 2019; Tang et al., 2019; Afolayan et al., 2020; Doumatey et al., 2020; Even et al., 2021; Parbie et al., 2021). The Prevotellacea family was the most frequently mentioned in 5 studies (Dugas et al., 2018; Afolayan et al., 2019; Hansen et al., 2019; Tang et al., 2019; Parbie et al., 2021) and is also mentioned to be the most prevalent in the African populations by Hansen et al. (2019).

#### Genera level

**Figure 3:**
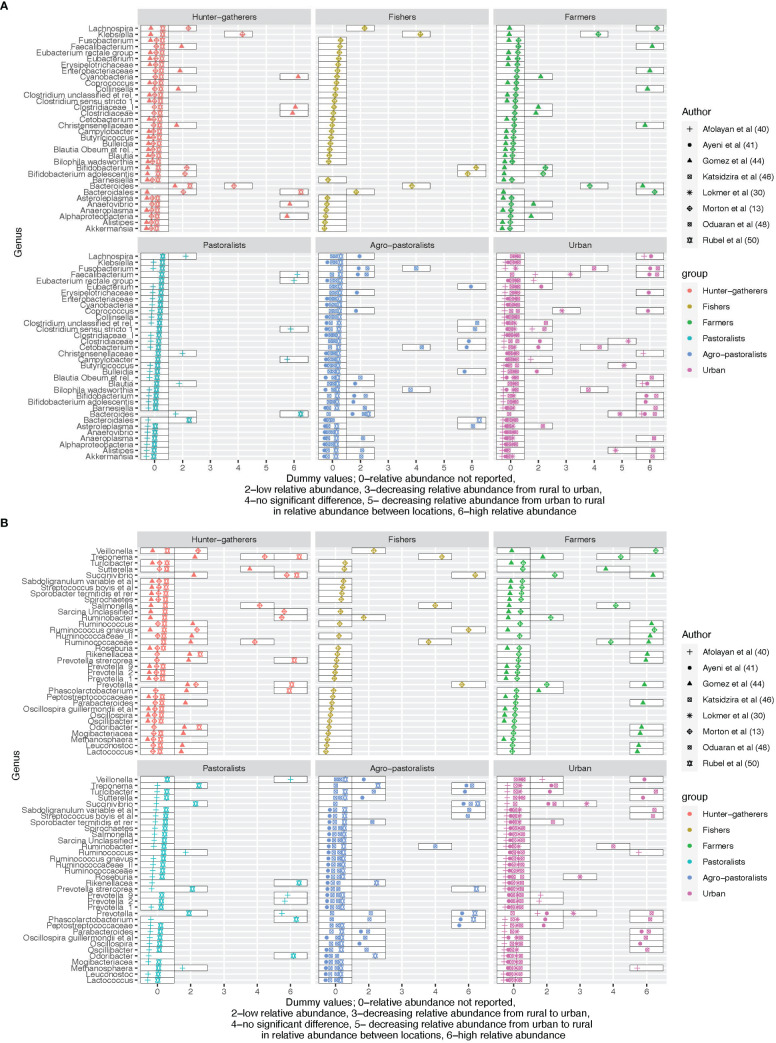
**(A)** An overview of the differences in relative abundance at genus level across the urbanization gradient for the first 34 reported genera. **(B)** An overview of the differences in relative abundance at genus level across the urbanization gradient for the last 34 reported genera.

Eight studies compared the relative abundance across hunter-gatherers, rural, semi-urban, or urban areas at the genus level as shown in **Figure 3**. Complex carbohydrate digesters *Bacteroides* were the most frequently reported and appeared in five studies (Morton et al., 2015; Afolayan et al., 2019; Lokmer et al., 2020; Oduaran et al., 2020; Rubel et al., 2020). The relative abundance of *Bacteroides* according to the urbanization level of the assessed population varied across studies. They had a higher relative abundance among hunter-gatherers and rural farmers (Morton et al., 2015), pastoralists (Rubel et al., 2020), and urban (Afolayan et al., 2019). In contrast, their relative abundances were low in hunter-gatherers (Rubel et al., 2020), rural fishers (Morton et al., 2015), pastoralists (Afolayan et al., 2019), and agro-pastoralists (Oduaran et al., 2020; Rubel et al., 2020) and decreased over the urbanization gradient from urban to rural (Lokmer et al., 2020). Hence, the direction of the relative abundance of *Bacteroides* does not follow a clear gradient. Similar to *Bacteroides*, there is no clear difference between urban and rural residents for *Bifidobacterium*, microorganisms known to digest carbohydrates and regulate gut health (Naumova et al., 2020). This component of the microbiota was compared across the urbanization gradient where it had a high relative abundance among rural fishers (Morton et al., 2015) and urban residents (Oduaran et al., 2020) but a low relative abundance among hunter-gatherers (Morton et al., 2015), and farmers and agro-pastoralists (Oduaran et al., 2020).

*Blautia* was more enriched among rural pastoralists and agro-pastoralists but low in urban (Ayeni et al., 2018; Afolayan et al., 2019) while *Blautia (species - obeum)* was high in urban and low among agro-pastoralists (Katsidzira et al., 2019). The relative abundance of *Bilophila (species - wadsworthia)* (linked with disrupted glucose metabolism in mice) (Natividad et al., 2018), *Cetobacterium*, *Ruminobacter*, and *Turicibacter* did not differ between rural agropastoralists and urban residents (Katsidzira et al., 2019; Oduaran et al., 2020).

The relative abundance of the *Treponema* genus was high in hunter-gatherers compared to rural and was also detected in urban (Oduaran et al., 2020) contrary to the findings in (Angelakis et al., 2019) where no *Treponema* was detected in urban areas. Out of the six named core genera, *Faecalibacterium* was the most frequently reported in the comparison (4 out of 8 studies), followed by *Blautia* (3 studies), *Clostridium* (2 studies), *Ruminococcus* (2 studies) and *Eubacterium* (1 study) (Dehingia et al., 2015). *Roseburia* was also reported in one study only where its relative abundance increased from rural to urban (Lokmer et al., 2020).

Similar to previously mentioned genera, *Faecalibacterium* did not follow a consistent pattern. It showed a positive correlation with the urbanization gradient from rural to urban (Lokmer et al., 2020), and displayed a high abundance among pastoralists (Afolayan et al., 2019) and urban (Ayeni et al., 2018; Oduaran et al., 2020), but a low abundance in agro-pastoralists (Ayeni et al., 2018; Oduaran et al., 2020) and urban residents (Afolayan et al., 2019). The relative abundance of *Fusobacterium* was similar for rural agro-pastoralists and urban residents (Katsidzira et al., 2019), low in agro-pastoralists but high in urban (Oduaran et al., 2020) and vice versa (Ayeni et al., 2018).

Carbohydrate fermenters and producers of the short-chain fatty acid butyrate (Ezaki, 2015), *Coprococcus* were more abundant in urban residents and low in agro-pastoralists (Ayeni et al., 2018) and increased with the urbanization gradient from rural to urban (Lokmer et al., 2020).

The genus *Prevotella* was reported in several studies including Prevotella, Prevotella_1, Prevotella_2, Prevotella_9, and Prevotella stercorea. In this review, *Prevotella* was more abundant in Hunter-gatherers (Gomez et al., 2016; Rubel et al., 2020), agro-pastoralists (Rubel et al., 2020), and urban (Oduaran et al., 2020). The genus decreased in relative abundance across the urbanization gradient from rural to urban (Lokmer et al., 2020) and was less abundant in rural farmers, pastoralists, and agro-pastoralists (Gomez et al., 2016; Oduaran et al., 2020; Rubel et al., 2020). Prevotella_1 was more abundant among hunter-gatherers (Gomez et al., 2016) and urban (Oduaran et al., 2020) and less abundant among farmers (Gomez et al., 2016) and agro-pastoralists (Oduaran et al., 2020). Prevotella_2 and Prevotella_9 were both abundant among pastoralists compared to urbanites (Afolayan et al., 2019) while Prevotella stercorea were more abundant among hunter-gatherers and low among pastoralists (Rubel et al., 2020). Refer to **Figure 3** for further details on the distribution of the relative abundance of the genera across the gradient.

#### The urbanization gradient and diet

Diet was assessed directly or indirectly in 11 studies using 24-hour recalls (Tang et al., 2019; Lokmer et al., 2020), food frequency questionnaires (FFQ) (Schnorr et al., 2014; Morton et al., 2015; Lokmer et al., 2020), narrative reports (Afolayan et al., 2019; Ocvirk et al., 2020), and nutritional questionnaires (Dugas et al., 2018; Rubel et al., 2020). Diets of urban residents consisted of dairy products, meat, sweets, and soft drinks (Lokmer et al., 2020) (Cameroon), low fiber-diet, processed foods, high-fiber diet, fermented drinks (Afolayan et al., 2019) (Nigeria), cereals, refined grains, fruits, and tubers, moderate use of antibiotics, use of treated water with a high level of hygiene (Ayeni et al., 2018) (Nigeria). In contrast, the rural residents reported consumption of tubers, leafy vegetables, peanuts, and palm wine (Lokmer et al., 2020) (Cameroon), grains, tubers, fruits and soups (okra, melon) and traditional soups, regular fish intake with less meat intake, a variety of fermented foods, use river water and rely on local herbs (Ayeni et al., 2018) and cassava, fish and meat (Morton et al., 2015) (Cameroon). Pastoralists reported eating high-fibre-based foods and fermented drinks while agro-pastoralists reported grain and vegetable consumption. Their livestock was mostly for selling purposes (Afolayan et al., 2019). Hunter-gatherers showed high-fiber diets and other food items from hunting and foraging (Rubel et al., 2020) (Cameroon), and high meat and nut consumption (Gomez et al., 2016) (Central Africa) but also low meat consumption (Morton et al., 2015) (Cameroon). Carbohydrate and fiber intake were observed to be similar in urban and rural settings with a high intake of protein in urban (Zimbabwe) (Katsidzira et al., 2019).

#### Diet and gut microbiota

The relationship between diet and gut microbiota was assessed in 4 studies only (Morton et al., 2015; Smits et al., 2017; Tang et al., 2019; Rubel et al., 2020). For the Hadza community (Hunter-gatherers) there was a strong link between seasonal changes in diet and gut microbiota composition (Smits et al., 2017). During the wet season, Hadza consumes a plant-based diet (berries, honey, baobab, and tubers) and has access to more meat during dry seasons. Consequently, there was a decline in the population of Bacteroidetes (Prevotellaceae family) were depleted during the wet season and reappeared in the dry season. In contrast, Firmicutes remained stable throughout the seasons.

Second, after adjusting for the presence of Entamoeba spp, Morton et al. (2015) found a persistent low gut microbiota diversity among the fishing community compared to hunter-gatherers and subsistence farmers. Lastly, the presence of genus *Ruminoccocus* was associated with meat and iron intake while a high relative abundance of *Faecalibacterium* and *Succininvibrio* was positively correlated with fish and/or insect intake and vitamin A-rich green vegetables and fruits (Tang et al., 2019). Further details are given in **Supplementary Material Appendix 3**.

### The relationship between the gut microbiota and intestinal parasites

**Figure 4:**
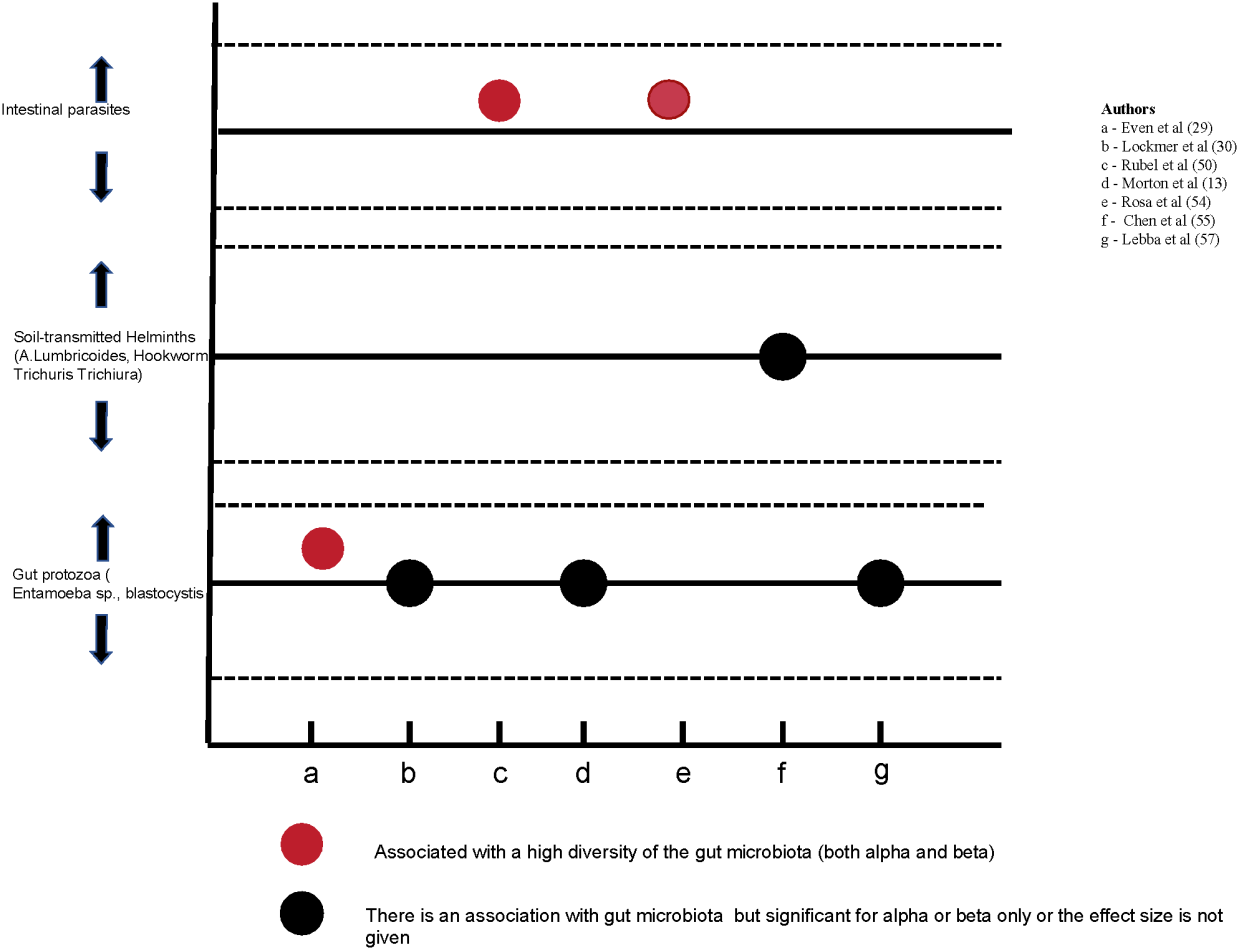
The relationship between the gut microbiota and intestinal parasites as reported in 7 studies.

The relationship between the gut microbiota and intestinal parasites was reported in seven studies (Morton et al., 2015; Iebba et al., 2016; Rosa et al., 2018; Lokmer et al., 2020; Rubel et al., 2020; Chen et al., 2021; Even et al., 2021). Details are given in **Figure 4** below. The presence of the intestinal parasites was assessed using microscopy in three studies (Morton et al., 2015; Lokmer et al., 2020; Chen et al., 2021), a combination of microscopy and quantitative PCR (qPCR) in two studies (Rosa et al., 2018; Rubel et al., 2020), qPCR in one study (Iebba et al., 2016) and 18S rRNA gene sequencing in one study (Even et al., 2021). The techniques used for the diagnosis of intestinal parasites complement each other as there is no gold standard (Maurelli et al., 2022). All studies that examined the relationship between intestinal parasites and gut microbiota showed that the presence of parasites is correlated with the diversity of the gut microbiota. Their presence was associated with a higher diversity of gut microbiota in two studies (Rubel et al., 2020; Even et al., 2021) but the direction of the association was not explicitly shown in the remaining 5 studies. In a longitudinal study, Rosa et al. (2018) found that gut microbiota alpha diversity was not fully restored two years after the administration of oral anti-helminths (**Figure 4**).

### The differences in the predicted functional profiles of the identified gut microbiota

To identify pathways and hierarchy, information obtained from the pipelines was collapsed using the Kyoto Encyclopedia of Genes and Genomes (KEGG) pathways and metabolites classification. Metabolic pathways were reported in 13 studies and were reported across the gradient in 5 studies (refer to **Supplementary Material Appendix 4**). Although most pathways are shared across the urbanization gradient differences in enrichment were reported in two studies (Afolayan et al., 2019; Hansen et al., 2019). In a study comparing urban and rural, methane metabolism, arginine and proline metabolism, pyruvate metabolism, valine, leucine, and isoleucine biosynthesis, and fructose and mannose metabolism were more enriched in urban residents (Afolayan et al., 2019). The same pathways are reported among hunter-gatherers and rural residents with no details on the differences in enrichment (Morton et al., 2015; Gomez et al., 2016; Dugas et al., 2018). Similarly, in a study conducted by Afolayan et al. as well as in other studies (**Supplementary Material Appendix 4**), lipopolysaccharide biosynthesis, ubiquinone and other terpenoid quinone synthesis, glycosyltransferases, arachidonic acid metabolism, riboflavin, vitamin B6 metabolism, protein digestion, and absorption were shown to be more enriched among rural residents (Afolayan et al., 2019). Finally, amino acid metabolism was reported throughout the urbanization gradient and no differences were reported in the pathway enrichment.

## Discussion

We reviewed 23 peer-reviewed publications that assessed the gut microbiota profile of healthy residents of 13 SSA countries across the urban-rural gradient. In addition to geographical differences the studies reported on the diet, the use of anti-helminths, intestinal parasites, and the functional potential of the identified pathways. All the included studies used Next Generation Sequencing techniques although the heterogeneity was high in sample collection, data processing including sequencing depth and platform, regions of the 16S gene sequenced, and downstream analysis like diversity and differential abundance analysis. Ten studies reported taxonomic levels and other differences related to the gut microbiota between geographical locations along the urbanization gradient (Schnorr et al., 2014; Gomez et al., 2016; Ayeni et al., 2018; Afolayan et al., 2019; Angelakis et al., 2019; Hansen et al., 2019; Katsidzira et al., 2019; Lokmer et al., 2020; Oduaran et al., 2020; Rubel et al., 2020).

### General trends of the gut microbiota studies as compared with non-SSA countries

#### Gut microbiota diversity

We observed a difference in both alpha and beta diversity across the urbanization gradient with a higher diversity in hunter-gatherers/rural compared to urban communities. However, the taxa level and diversity metric analysis used differed between studies. The results we found are similar to a meta-analysis on the impact of urbanization on the gut microbiota globally (including a subset of the same studies from SSA included in this review) that showed a lower alpha diversity in urban populations compared to rural/hunter-gatherers settings (Rosas-Plaza et al., 2022). In this meta-analysis beta diversity at family and genera, levels showed similar trends, with the urban and Hunter-gatherers communities at the extremes (lowest vs highest diversity) (Rosas-Plaza et al., 2022). Several studies have evaluated this in other parts of the world with varying results (Clemente et al., 2015; Das et al., 2018; Jha et al., 2018; McCall et al., 2020; Rosas-Plaza et al., 2022). For example, a study in Tibet China showed differences in beta diversity and not alpha diversity across the urbanization gradient, contrary to the studies included here with differences in both measures (Li et al., 2018). Likewise, a study conducted among populations transitioning from foragers to farmers in the Himalayan region did not find any variations in alpha diversity (Jha et al., 2018). Conversely, a study done in South America, which explored the overall and chemical variation across the spectrum from traditional villages to the city center, did not find any differences in diversity along the urbanization gradient (McCall et al., 2020). In general, evidence indicates that gut microbiota in higher-income/industrialized exhibit lower diversity compared to lower-income/non-industrialized/rural/traditional communities (Obregon-Tito et al., 2015; Mancabelli et al., 2017; Rosas-Plaza et al., 2022). Nevertheless, there is a need for additional studies to fully understand the distinctions in gut microbiota among individuals residing in geographically close areas.

#### Reported specific phylum/genera, differential abundance, biological effects, and associated health outcomes

Bacteroidetes and Firmicutes are known to dominate the gut microbiota. Six other phyla are proposed to form the 8 core gut microbiota by the healthy gut microbiota (GutFeelingKB data and metadata); Proteobacteria, Actinobacteria, Spirochaetes, Plancytomycetes, Euryarchaeota and Cyanobacteria (King et al., 2019). Similar, to the above-established core gut microbiota, Firmicutes and Bacteroidetes were the most abundant phyla in the included studies. Further, there are differences in the relative abundance of gut microbiota across the urbanization gradient although not uniform for all the phyla/genera as some were relatively more abundant while others were less abundant, as shown in **Figure 2**.

The microbiome of the rural (Hunter-gatherers/traditional communities and non-urban) is associated with a low prevalence of diseases including inflammatory bowel diseases, colorectal cancer, and metabolic diseases (Zuo et al., 2018; Cui et al., 2021). Perturbations in a few phyla have been linked to health outcomes such as the Firmicutes: Bacteroidetes ratio where higher Bacteroidetes levels are seen in lean compared to obese individuals (Johnson et al., 2017). Also, a higher relative abundance of Bacteroidetes and Proteobacteria compared to Firmicutes was reported among patients with chronic kidney disease (Lun et al., 2019). In this review, the ratio (Firmicutes: Bacteroidetes) was reported in one study only where it was 5:1 in rural and 1:1 in the Hunter-gatherers’ community (Gomez et al., 2016).

A study in South America from the jungle to the city center by McCall et al. showed a high proportion of Proteobacteria with increasing urbanization (McCall et al., 2020). Similarly, in this review, the relative abundance of Proteobacteria was higher in rural compared to hunter-gatherers’ settings. In contrast to the findings in South America, where the relative abundance of Actinobacteria decreased with urbanization (higher in hunter-gatherers), our review did not identify any clear trend for this phylum (McCall et al., 2020).

Most studies reported the gut microbiota differences at the genus level. Existing evidence has linked individual genera to disease conditions and health outcomes. In this review, there are no clear differences in the relative abundance of identified genera across the urbanization gradient except for *Coprococcus* where the relative abundance was consistently low in rural and higher in urban (Ayeni et al., 2018; Lokmer et al., 2020). *Bacteroides* were the most frequently reported in the studies that compared the relative abundance across the gradient. They are known to maintain gut health, digest complex carbohydrates, metabolize bile, maintain the immune system and gut barrier, and produce Vitamins K and B (Xu and Gordon, 2003). This genera together with *Faecalibacterium* and *Blautia* were more abundant in urban herdsmen in Tibet contrary to the findings of this review where their relative abundance did not follow a clear gradient (Li et al., 2018).

The relative abundance of the *Lactobacillus* genus was reported to be high among the Mongolian pastoralists compared to the urban Mongolians possibly due to the high consumption of dairy products (Iebba et al., 2016). However, in our review, *Lactobacillus* was reported in two rural communities (Iebba et al., 2016; Tang et al., 2019) and there was no difference between pastoral and non-pastoral communities (Afolayan et al., 2019). The biological importance of Lactobacillus is not fully understood as their relative abundance is both negatively and positively correlated with disease outcomes (Heeney et al., 2018).

A study assessing the gut microbiota based on diet, ethnicity, and urbanization in China identified four types of enterotypes including one dominated by the *Escherichia* genus (Lu et al., 2021). This genus was also reported in a study in Korea contrasting gut microbiota between rural villages and urban (Wu et al., 2011). However, in this review, the *Escherichia* genus was not reported in any of the studies that compared gut microbiota across the gradient.

The relative abundance of the genus *Prevotella* varied across the urbanization gradient with a high and low relative abundance of the genera across the gradient without clear direction. *Prevotella* and its sub-genera were reported across the gradient with alternating relative abundances. The abundance of the *Prevotella* genus has been shown to correlate with carbohydrate consumption, which is documented in the included studies across the urbanization gradient (Sonnenburg et al. ). A higher relative abundance of Prevotella is often associated with plant-based diets and improved glucose metabolism (Kovatcheva-Datchary et al., 2015; Ley, 2016). The presence of Prevotella throughout the urbanization gradient indicates a potential trend between urbanization and industrialization, as the abundance of *Prevotella* is nearly absent in industrialized societies (Ley, 2016). This observation suggests that industrialization may have a greater effect on the abundance of this genus compared to the process of urbanization seen in SSA.

The composition and relative abundance of *Bifidobacterium* in mice fed with fish oil were higher compared to those eating lard (David et al., 2014). In line with this finding, this review found *Bifidobacterium* to have a high relative abundance among the fishing and urban communities compared to hunter-gatherers and agro-pastoralists (Oduaran et al., 2020). *Bifidobacterium* has been used as an important probiotic in several food products including yogurt and supplements and is believed to enhance a healthy gut (Naumova et al., 2020).

*Bilophila wadsworthia* is known to digest taurine a naturally occurring compound in meat and milk fat and produce hydrogen sulfide an inflammatory agent (Yazici et al., 2017; Hanson et al., 2021). Also, their ability to produce secondary bile is linked with the occurrence of colorectal cancer (Turnbaugh, 2012). The relative abundance of *Bilophila wadsworthia* did not differ between rural agro-pastoralists and urban residents (Katsidzira et al., 2019; Oduaran et al., 2020). This may signify a similarity in animal-based diet consumption among rural agro-pastoralists and urban residents (Mehta et al., 2017).

*Blautia* was reported to be low among rural pastoralists and agro-pastoralists but high in urban (Ayeni et al., 2018; Afolayan et al., 2019) while *Blautia Obeum* was high in urban and low among agro-pastoralists (Katsidzira et al., 2019). In Kim et al, the relative abundance of *Blautia* was low in urban Korea compared to rural villages (Wu et al., 2011). This genus is associated with better host health and reduced risk of metabolic syndrome (Liu et al., 2021).

*Coprococcus*, a butyrate producer, is positively correlated with a high-quality diet and negatively correlated with hypertension (Mancabelli et al., 2017; Laitinen and Mokkala, 2019). Two studies found *Coprococcus* were more abundant in urban residents and low in agro-pastoralists (Ayeni et al., 2018) and increased with the urbanization gradient from rural to urban (Lokmer et al., 2020). This is contrary to a study in Japan where Coprococcus were least abundant in urban (Naito et al., 2019). Further, without following the urbanization gradient, the *Coprococcus* genus was found to dominate the Brazilian population compared to Cameroonians (Schaan et al., 2021).

*Fusobacterium* is an important gut commensal organism that plays a key role in protecting the body from pathogens (Brennan and Garrett, 2019; Stokowa-Sołtys et al., 2021). However, its isolation from disease-related samples (clinical) has indicated a potential role in disease causation and progression questioning the direction of its effect on health (Brennan and Garrett, 2019). *Fusobacterium* is linked to an increased risk of colorectal cancer and its high relative abundance is associated with the consumption of Westernized diets (Mehta et al., 2017). In this review, the distribution of *Fusobacterium* was mixed with some studies reporting high relative abundance in urban areas while others reported a low abundance or no difference between urban and rural residents (Katsidzira et al., 2019; Afolayan et al., 2020; Oduaran et al., 2020).

Similar to *Fusobacterium, Faecalibacterium* strengthens the gut mucosal integrity and maintains the health of the gut and organism physiology through the production of different anti-inflammatory metabolites such as butyrate and salicylic acid (He et al., 2021). It is recognized as the most ubiquitous species due to its presence in the gut of most mammals including humans and was similarly the most frequently reported genus in this review (He et al., 2021). *Faecalibacterium* was more abundant in the mountainous rural compared to urbanizing and urbanized areas in Ningxia China (Du et al., 2021). We did not find a clear gradient for *Faecalibacterium* in the included studies. Like Fusobacterium, F*ecalibacterium* also exhibits a bidirectional impact on health, with a low relative abundance being associated with irritable bowel syndrome and a high relative abundance being linked to colorectal cancer (Rajilić-Stojanović et al., 2011; Keku et al., 2013).

#### Diet and gut microbiota

Diets and individual foods influence the diversity and composition of the gut microbiota as shown in several reviews (Frame et al., 2019; Willis and Slavin, 2020; Kimble et al., 2022; Wang et al., 2022). A scoping review of randomized controlled trials on the effects of meat on the gut microbiota showed an increase in *Ruminococcus, Roseburia, Bacteroides*, and *Anaerostipes* genera with a high intake of meat while *Faecalibacterium* decreased (Wang et al., 2022). A review of the adherence to the Mediterranean diet and gut microbiota did not show any relationship at the higher taxonomic level (phyla) but at the genera level with *Ruminococcus, Bacteroides*, and *Faecalibacterium* (Kimble et al., 2022). Another systematic review of the effect of fiber supplementation on gut microbiota showed a significantly higher relative abundance of *Bifidobacterium* and *Lactobacillus* compared to other genera (So et al., 2018).

In this review, we could not conclude on the differences in diets across the urbanization gradient and its effect on the gut microbiota as only a few studies used standard methods of dietary assessment. One study reported the link between diet and gut microbiota where similar to (Wang et al., 2022) *Ruminococcus* was linked to meat and iron intake and *Faecalibacterium* to fish, Vitamin A-rich green vegetables and fruits similar to (Kimble et al., 2022). Also, there was evidence of changes in the gut microbiota in response to changing diets and seasons. This phenomenon was described in one study only and not across the gradient. Hence, it is evident that the effects of diet on the diversity and composition of the gut microbiota are still under-researched in the SSA. Included studies have mostly named the macronutrients without further quantification of the consumed diets, the micronutrient contents, or the analysis of dietary patterns. For example, the high-fiber diets reported in both rural and urban communities may differ significantly in quantity and quality hence their effect on the gut microbiota.

#### Intestinal parasites and gut microbiota

Intestinal parasites are associated with the composition and diversity of the gut microbiota. Their low prevalence in industrialized communities is thought to contribute to the lower diversity of the gut microbiota, although the mechanism underlying this association is poorly understood (Chabé et al., 2017). A study in Malaysia showed a higher microbial diversity among helminths positive compared to negative colonized individuals (Lee et al., 2014). The evidence from this review partly affirms this finding as it shows a relationship between gut microbiota and intestinal parasites. The presence of intestinal parasites was associated with a high diversity of the gut microbiota in two studies (Rubel et al., 2020; Even et al., 2021) but the effect size was not shown in some studies. Further, the use of anti-helminths was linked to a long-term loss of gut microbiota diversity (Rosa et al., 2018). As the relationship between gut microbiota and intestinal parasites is increasingly seen further studies to inform the public health efforts of mass deworming are needed. The absence of intestinal parasites has also been linked to an increased risk of inflammatory bowel diseases and Crohn’s disease (Panelli et al., 2020).

#### Health implications of the reported functional profile of the identified gut microbiota

Evidence links metabolites and enriched metabolic pathways of the gut microbiota with several negative and positive health outcomes. Metabolites and pathways, such as the production of short-chain fatty acids in the colon, amino acid metabolism, carbohydrate metabolism, and secondary metabolite biosynthesis pathways, have been shown to positively influence health outcomes (Lin et al., 2017). Gut microbiome metabolites such as trimethylamine N-oxide (TMAO) and hydrogen sulfide are thought to affect gut and cardiovascular health in both positive and negative ways (Wu et al., 2021). However, evidence is still building on other gut microbiome by-products such as “p-cresol” being a potential neurotoxic (Tevzadze et al., 2017; Tevzadze et al., 2018). In this review, differences in pathway enrichment between rural and urban were reported in two studies where amino acid and carbohydrate metabolism pathways were enriched in the urban population (Afolayan et al., 2019) while carbohydrate metabolism gene pathways were depleted in the hunter-gatherers population (Gomez et al., 2016). The differences in the enrichment were not stated in most studies, however, we noted a uniform reporting of the amino acid metabolism pathways.

Low dietary protein intake is linked with the body using essential amino acids produced by the gut microbiome (Wu et al., 2021). Some bacteria species (e.g. *Clostridium acetobutylicum*) are known to have a complete gene set for essential amino acids biosynthesis (Nolling et al., 2001). Hence, the wide reporting of the amino acid metabolism pathways may signify a diet lacking all the essential amino acids by the included population. However, given the complex relationship and interpretation of metabolic pathways other factors may explain the pattern of the reported amino acid metabolism pathways. A study done in China in two adjacent rural and urban provinces reported 266 out of 1108 differences in metabolites between the two sites (Wang et al., 2020). Six metabolites were twice the amount in urban compared to rural for Xanthine metabolism (derived from caffeine) (Wang et al., 2020). Other metabolites and pathways enriched in urban included those causing inflammation and metabolic derangements (Wang et al., 2020). Characterization of the gut microbiome metabolic pathways and endogenous and exogenous metabolites is still a growing field. Thus, we did not find a large body of evidence comparing the differences along the urbanization gradient to draw robust inferences.

### Limitations and future directions

In addition to external perturbation, the gut microbiota is sensitive to several individual factors including physical activity levels, household, and lifestyle factors such as smoking, alcohol intake, occupation, economic levels, and cultural beliefs (Xu and Gordon, 2003). The assessment of these factors with the gut microbiota was not explicitly featured in the included studies. Evidence from McCall et al. shows the differences in the gut microbiota diversity are partly explained by the individual’s socioeconomic levels and not their location along the urbanization gradient (McCall et al., 2020). Further, we noted a variation in the definition of urbanization gradient as disparate approaches were used across studies which limited inter-study comparison. In some studies, the definition was based on subsistence modes while others considered geographical locations or developed indices. Multiple factors related to urbanization are known to impact gut microbiota, therefore, it is important to develop and use standard approaches for unified assessments. In this instance, indices to measure the urbanization gradient as done by Lokmer et al. and adapting standard approaches in the dietary assessment such as the 24-hour recall and Food frequency questionnaires (FFQ) should be considered (Lokmer et al., 2020).

The methods for sample collection, DNA extraction, and analysis varied significantly among the studies. Although alpha and beta diversities were often reported, the reporting was not consistent. In general, the results suggested the diversity of the gut microbiota differed across the urbanization gradient. Thus, the comparability of the studies was limited by heterogeneity in sequencing, analytical approaches, and reporting. Several initiatives propose approaches to standardizing methodologies and reporting microbiome studies (Amos et al., 2020; Mirzayi et al., 2021). Hence, upcoming studies should strive to incorporate these standards for robust comparisons across different studies and datasets. Standardization approaches, similar to those used in the study of microbiome should be considered in the investigation of the determinants of the gut microbiota.

The included studies had several limitations which affected the inter-study comparisons. Statistically, most studies did not give the sample selection strategy and rationale. Secondly, estimated sample sizes were justified in two studies only and there was a remarkable variation in the sample sizes that ranged from 10 to 575. Most studies were insufficiently powered to detect statistical differences. In this context, we assume most study samples were influenced by high sequencing costs although not evident in all studies. As the costs of sequencing decrease and the quality of sequencing technologies improve, larger sample sizes and studies beyond cross-sectional studies should be considered. These may include longitudinal studies to document population changes in gut microbiota including case-crossover studies that follow people at the time when they move or undergo diet transitions.

## Conclusion

This review aimed to examine the dissimilarities in the composition and diversity of gut microbiota in the transitioning SSA region. Generally, the findings indicate that there are variations in the gut microbiota diversity between urban and rural/traditional communities, although the evidence regarding specific taxa levels for transitioning communities is inconsistent. In comparison to traditional/rural communities, urban communities exhibited lower diversity in their gut microbiota, while certain rural communities (pastoralists) show decreased diversity. Therefore, the urbanization process in the SSA is associated with the loss of diversity, but there is insufficient evidence to indicate changes in the composition of the gut microbiota. The lower diversity of the gut microbiota may be a driving factor in increasing NCDs in transitioning communities in SSA.

## Data availability statement

The original contributions presented in the study are included in the article/**Supplementary Material**, further inquiries can be directed to the corresponding author/s.

## Author contributions

The research question was conceptualized by VL, FA, and LP. LP and GB independently screened abstracts and extracted study findings. LP analyzed and synthesized extracted data, supported by VL. LP wrote and revised the manuscript, incorporating comments from VL and other authors KK-G, FA, PC, AK, GK, and GB. All authors contributed to the article and approved the submitted version.
