# Supplementary material for: Urbanization gradient, diet, and gut microbiota in Sub-Saharan Africa: a systematic review
Source: Front Microbiomes. 2023 Sep 12;2:1208166. doi: 10.3389/frmbi.2023.1208166 (PMC12993604; doi:10.3389/frmbi.2023.1208166)
Supplement: Supplementary file 1 [file DataSheet_1.pdf]

Supplementary Material

Urbanization gradient, diet, and gut microbiota in Sub-Saharan Africa: A systematic review

Linda Simon Paulo[1,2*], George Msema Bwire[2,10], K. Klipstein-Grobusch[1,3,4], Appolinary Kamuhabwa[2,9], Gideon Kwesigabo[2,5], Pilly Chillo[2,8], Folkert W. Asselbergs[6,7], Virissa C. Lenters[1]

[1] Department of Epidemiology and Global Health, Julius Center for Health Sciences and Primary Care, University Medical Center Utrecht, Utrecht University, Utrecht, the Netherlands

[2] Cardiac Center of Excellence, Muhimbili University of Health and Allied Sciences, Tanzania

[3] Division of Epidemiology and Biostatistics, School of Public Health, Faculty of Health Sciences, University of the Witwatersrand, Johannesburg, South Africa

[4] Institute for Tropical Medicine, University of Tübingen, Tübingen, Germany

[5] Department of Biostatistics and Epidemiology, School of Public Health and Social Sciences, Muhimbili University of Health and Allied Sciences, Tanzania

[6] Amsterdam University Medical Centers, Department of Cardiology, University of Amsterdam, Amsterdam, The Netherlands

[7] Health Data Research UK and Institute of Health Informatics, University College London, London, United Kingdom

[8] Department of Cardiology, Jakaya Kikwete Cardiac Institute, Muhimbili, Dar es Salaam, Tanzania

[9] Department of Clinical Pharmacy and Pharmacology, School of Pharmacy, Muhimbili University of Health and Allied Sciences, Tanzania

[10] Department of Pharmaceutical Microbiology, School of Pharmacy, Muhimbili University of Health and Allied Sciences, Tanzania

***Correspondence:**
Linda Simon Paulo
Linda.p.simon@gmail.com

1 Appendix 1

A list of search strategies used;

PubMed

1. ((((((((gastrointestinal micro*[Title/Abstract]) OR (gut flora[Title/Abstract])) OR (intestinal micro*[Title/Abstract])) OR (gut micro*[Title/Abstract])) OR (intestinal flora[Title/Abstract])) OR (enteric bacteri*[Title/Abstract])) OR (gastric micro*[Title/Abstract])) OR ("Gastrointestinal Microbiome"[Mesh]))
2. microbiota OR microbiome
3. Angola*[tw] OR Benin*[tw] OR Botswan*[tw] OR Burkina Faso*[tw] OR Burundi*[tw] OR Cabo Verde*[tw] OR Cape Verde*[tw] OR Cameroon*[tw] OR Central Africa*[tw] OR Chad*[tw] OR Comoro*[tw] OR Congo*[tw] OR Cote d'Ivoire*[tw] OR Djibouti*[tw] OR East Africa*[tw] OR Eastern Africa*[tw] OR Equatorial Guinea*[tw] OR Eritre*[tw] OR Ethiopia*[tw] OR Gabon*[tw] OR Gambia*[tw] OR Ghan*[tw] OR Guinea*[tw] OR Ivory Coast*[tw] OR Kenya*[tw] OR Lesotho*[tw] OR Liberia*[tw] OR Madagascar*[tw] OR Malaw*[tw] OR Mali[tw] OR Mauritan*[tw] OR Mauriti*[tw] OR Mocambiqu*[tw] OR Mozambiqu*[tw] OR Namibia*[tw] OR Niger*[tw] OR Rhodesia*[tw] OR Rwanda*[tw] OR Sao tome and Principe*[tw] OR Senegal*[tw] OR Seychelle*[tw] OR Sierra Leone*[tw] OR Somalia*[tw] OR South Africa*[tw] OR South Sudan*[tw] OR Southern Africa*[tw] OR sub Saharan Africa*[tw] OR subSaharan Africa*[tw] OR Sudan*[tw] OR Swaziland*[tw] OR Tanzan*[tw] OR Togo*[tw] OR Ugand*[tw] OR West Africa*[tw] OR Western Africa*[tw] OR Zaire*[tw] OR Zambia*[tw] OR Zimbabw*[tw] OR Angola*[ad] OR Benin*[ad] OR Botswan*[ad] OR Burkina Faso*[ad] OR Burundi*[ad] OR Cameroon*[ad] OR Cabo Verd*[ad] OR Cape Verd*[ad] OR Central Africa*[ad] OR Chad*[ad] OR Comoro*[ad] OR Congo*[ad] OR Cote d'Ivoire*[ad] OR Djibouti*[ad] OR East Africa*[ad] OR Eastern Africa*[ad] OR Equatorial Guinea*[ad] OR Eritre*[ad] OR Ethiopia*[ad] OR Gabon*[ad] OR Gambia*[ad] OR Ghan*[ad] OR Guinea*[ad] OR Ivory Coast*[ad] OR Kenya*[ad] OR Lesotho*[ad] OR Liberia*[ad] OR Madagascar*[ad] OR Malaw*[ad] OR Mali[ad] OR Mauritan*[ad] OR Mauriti*[ad] OR Mocambiqu*[ad] OR Morocc*[ad] OR Mozambiqu*[ad] OR Namibia*[ad] OR Niger*[ad] OR Rhodesia*[ad] OR Rwanda*[ad] OR Sao tome and Principe*[tw] OR Senegal*[ad] OR Seychelle*[ad] OR Sierra Leone*[ad] OR Somalia*[ad] OR South Africa*[ad] OR South Sudan*[ad] OR Southern Africa*[ad] OR Sri Lanka*[ad] OR sub Saharan Africa*[ad] OR subSaharan Africa*[ad] OR Sudan*[ad] OR Swaziland*[ad] OR Tanzan*[ad] OR Togo*[ad] OR Ugand*[ad] OR West Africa*[ad] OR Western Africa*[ad] OR Zaire*[ad] OR Zambia*[ad] OR Zimbabw*[ad]
4. urban*[Title/Abstract]) OR ("Urbanization"[Mesh]
5. #1 OR #2
6. #5 AND #3
7. #6 AND #4

Web of Science

1. Microbiota OR microbiome
2. Angola* OR Benin* OR Botswan* OR Burkina Faso* OR Burundi* OR Cabo Verde* OR Cape Verde* OR Cameroon* OR Central Africa* OR Chad* OR Comoro* OR Congo* OR Cote d'Ivoire* OR Djibouti* OR East Africa* OR Eastern Africa* OR Equatorial Guinea* OR Eritre* OR Ethiopia* OR Gabon* OR Gambia* OR Ghan* OR Guinea* OR Ivory Coast* OR Kenya* OR Lesotho* OR Liberia* OR Madagascar* OR Malaw* OR Mali OR Mauritan* OR Mauriti* OR Mocambiqu* OR Mozambiqu* OR Namibia* OR Niger* OR Rhodesia* OR Rwanda* OR Sao tome and Principe* OR Senegal* OR Seychelle* OR Sierra Leone* OR Somalia* OR South Africa* OR South Sudan* OR Southern Africa* OR sub Saharan Africa* OR subSaharan Africa* OR Sudan* OR Swaziland* OR Tanzan* OR Togo* OR Ugand* OR West Africa* OR Western Africa* OR Zaire* OR Zambia* OR Zimbabw*
3. #1 AND #2
4. urban* or urbanization
5. #3 AND #4

Scopus

1. {gastrointestinal micro*} OR {gut flora} OR {intestinal micro*} OR {gut micro*} OR {intestinal flora} OR {enteric bacteri*} OR {gastric micro*} OR {Gastrointestinal Microbiome}
2. microbiota OR microbiome
3. Angola* OR Benin* OR Botswan* OR {Burkina Faso*} OR Burundi* OR {Cabo Verde*} OR {Cape Verde*} OR Cameroon* OR {Central Africa*} OR Chad* OR Comoro* OR Congo* OR {Cote d'Ivoire*} OR Djibouti* OR {East Africa*} OR {Eastern Africa*} OR {Equatorial Guinea*} OR Eritre* OR Ethiopia* OR Gabon* OR Gambia* OR Ghan* OR Guinea* OR {Ivory Coast*} OR Kenya* OR Lesotho* OR Liberia* OR Madagascar* OR Malaw* OR Mali OR Mauritan* OR Mauriti* OR Mocambiqu* OR Mozambiqu* OR Namibia* OR Niger* OR Rhodesia* OR Rwanda* OR {Sao tome and Principe*} OR Senegal* OR Seychelle* OR {Sierra Leone*} OR Somalia* OR {South Africa*} OR {South Sudan*} OR {Southern Africa*} OR {sub Saharan Africa*} OR {subSaharan Africa*} OR Sudan* OR Swaziland* OR Tanzan* OR Togo* OR Ugand* OR {West Africa*} OR {Western Africa*} OR Zaire* OR Zambia* OR Zimbabw*
4. #1 AND #3
5. #2 AND #3

Embase

1. #1 'microbiome'/exp OR 'intestine flora'/exp
2. #2 'Angola'/exp OR 'Angolan'/exp OR 'Benin'/exp OR 'Beninese'/exp OR 'Botswana'/exp OR 'tswana people'/exp OR 'Burkina Faso'/exp OR 'Burkinabe'/exp OR 'Burundi'/exp OR 'Cape Verde'/exp OR 'Cameroon'/exp OR 'Cameroonian'/exp OR 'Central Africa'/exp OR 'Chad'/exp OR 'Chadic people'/exp OR 'Comoros'/exp OR 'Congo'/exp OR 'congolese kinshasa'/exp OR 'congolese brazzaville'/exp OR 'Cote d`Ivoire'/exp OR 'Djibouti'/exp OR 'Africa'/exp OR 'African'/exp OR 'Equatorial Guinea'/exp OR 'Equatorial Guinean'/exp OR 'Eritrea'/exp OR 'Eritrean'/exp OR 'Ethiopia'/exp OR 'Ethiopian'/exp OR 'Gabon'/exp OR 'Gabonese'/exp OR 'Gambia'/exp OR

'Gambian'/exp OR 'Ghana'/exp OR 'Ghanaian'/exp OR 'Guinea'/exp OR 'Guinea-Bissau'/exp OR 'Cote d`Ivoire'/exp OR 'Kenya'/exp OR 'Kenyan'/exp OR 'Lesotho'/exp OR 'Liberia'/exp OR 'Liberian'/exp OR 'Madagascar'/exp OR 'Malawi'/exp OR 'Malawian'/exp OR 'Mali'/exp OR 'Malian'/exp OR 'Mauritania'/exp OR 'Mauritanian'/exp OR 'Mauritius'/exp OR 'Mauritian'/exp OR 'Mozambique'/exp OR 'Mozambican'/exp OR 'Namibia'/exp OR 'Namibian'/exp OR 'Niger'/exp OR 'Niger-Congo people'/exp OR 'Nigeria'/exp OR 'Nigerian'/exp OR 'Zimbabwe'/exp OR 'Zimbabwean'/exp OR 'Rwanda'/exp OR 'Rwandan'/exp OR 'Sao Tome and Principe'/exp OR 'Sao Tome and Principe'/exp OR 'Senegal'/exp OR 'Senegalese'/exp OR 'Seychelles'/exp OR 'seychellene'/exp OR 'Sierra Leone'/exp OR 'Sierra Leonean'/exp OR 'Somalia'/exp OR 'somali citizen'/exp OR 'somali people'/exp OR 'South Africa'/exp OR 'South African'/exp OR 'South Sudan'/exp OR 'Sudanese'/exp OR 'Sudanese'/exp OR 'Africa south of the Sahara'/exp OR 'Eswatini'/exp OR 'Sudan'/exp OR 'Tanzania'/exp OR 'Tanzanian'/exp OR 'Togo'/exp OR 'Togolese'/exp OR 'Uganda'/exp OR 'Ugandan'/exp OR 'west africa'/exp OR 'West African'/exp OR 'Democratic Republic Congo'/exp OR 'Zambia'/exp OR 'Zambian'/exp

3. #3 'urbanization'/exp OR 'urbanization changes'/exp
4. #1 AND #2 AND #3

Table 2A: Assessment of risk of bias using the New-Castle Ottawa (NOS) for the case-control and cross-sectional studies

	**Selection(Maximum 5 points)**				**Comparability: (Maximum 2 points)**	**Outcome: (Maximum 3 points)**		**Total score (out of 10)**	
**Author**	**Representativeness of the sample**	**Sample size representativeness**	**Non-respondents**	**Ascertainment of the exposure**	**Design or analysis**	**Ascertainment of the outcome**	**Statistical test**		Risk of bias
Smits et al, 2017 (52)	0	0	0	2	0	2	1	5	Moderate
Schnorr et al 2013 (51)	0	0	0	2	1	2	1	6	Moderate
Gomez et al, 2016 (44)	0	0	0	2	1	2	1	6	Moderate
Angelakis et al, 2019 (56)	0	0	0	2	1	2	1	6	Moderate
Morton et al, 2015 (13)	0	0	0	2	1	2	1	6	Moderate
Rubel et al, 2020 (50)	0	0	0	2	1	2	1	6	Moderate
Hansen et al, 2019 (45)	0	0	0	2	1	2	1	6	Moderate
Chen et al, 2021 (55)	0	0	0	2	0	2	1	5	Moderate
Dugas et al, 2018 (42)	0	0	0	2	1	2	1	6	Moderate
Ellis et al, 2013 (43)	0	0	0	2	1	2	1	6	Moderate
Tang et al, 2019 (53)	0	0	0	2	1	2	1	6	Moderate
Rosa et al,2018 (54)	0	1	0	2	1	2	1	7	Low
Even et al,2021 (29)	0	0	0	2	1	2	1	6	Moderate
Lokmer et al,2020 (30)	0	0	0	2	1	2	1	6	Moderate
Parbie et al, 2021 (49)	0	0	0	2	1	2	1	6	Moderate
Katsidzira et al, 2019 (46)	1	1	0	2	1	2	1	8	Low
Ocvirk et al, 2020 (47)	0	0	0	2	1	2	1	6	Moderate
Oduaran et al, 2020 (48)	0	0	0	2	1	2	1	6	Moderate
Ayeni et al, 2018 (41)	0	0	0	2	0	2	1	5	Moderate
Lebba et al, 2016 (57)	0	0	0	2	1	2	1	6	Moderate
Afolayan et al, 2019 (40)	0	0	0	2	1	2	1	6	Moderate
Afolayan et al, 2020 (39)	0	0	0	2	1	2	1	6	Moderate
Doumatey et al, 2020* (20)	1	1	1	1	1	1	2	8	Low
* - case control study									
**Total scores ranking scale**: 0-3 – High, 4-6 – Moderate, 7-10 – Low									

Table 2B: Additional explanations to the scores in appendix 2A

	Sample and sample size selection (Max- 5 points)								Comparability: (Maximum 2 points)		Outcome: (Maximum 3 points)				
**Author, year**	**Sampling strategy**	**score**	**Sample size and representativeness**	**score**	**Non-respondents**	**score**	**Ascertainment of the exposure (risk factor)**	**score**	**The subjects in different outcome groups are comparable, based on the study design or analysis.**	**score**	**Assessment of the outcome**	**score**	**Statistical test:**	**score**	**Total score**
Smits et al, 2017 (52)	No description of the sampling strategy	0	Not justified.	0	No description of the response rate or the characteristics of the responders and the non-responders	0	Validated measurement tool. **	2	The study controls for the most important factor. *	0	Independent blind assessment. **	2	The statistical test used to analyze the data is clearly described and appropriate, and the measurement of the association is presented, including confidence intervals and the probability level (p value). *	1	5
Schnorr et al 2013 (51)	No description of the sampling strategy	0	Not justified.	0	No description of the response rate or the characteristics of the responders and the non-responders	0	Validated measurement tool. **	2	The study controls for the most important factor. *	1	Record linkage. **	2	The statistical test used to analyze the data is clearly described and appropriate, and the measurement of the association is presented, including confidence intervals and the probability level (p value). *	1	6
Gomez et al, 2016 (44)	No description of the sampling strategy	0	Not justified.	0	No description of the response rate or the characteristics of the responders and the non-responders	0	Validated measurement tool. **	2	The study controls for the most important factor. *	1	Record linkage. **	2	The statistical test used to analyze the data is clearly described and appropriate, and the measurement of the association is presented, including confidence intervals and the probability level (p value). *	1	6
Angelakis et al, 2019 (56)	No description of the sampling strategy	0	Not justified.	0	No description of the response rate or the characteristics of the responders and the non-responders	0	Validated measurement tool. **	2	The study controls for the most important factor. *	1	Record linkage. **	2	The statistical test used to analyze the data is clearly described and appropriate, and the measurement of the association is presented, including confidence intervals and the probability level (p value). *	1	6
Morton et al, 2015 (13)	No description of the sampling strategy	0	Not justified.	0	No description of the response rate or the characteristics of the responders and the non-responders	0	Validated measurement tool. **	2	The study controls for the most important factor. *	1	Record linkage. **	2	The statistical test used to analyze the data is clearly described and appropriate, and the measurement of the association is presented, including confidence intervals and the probability level (p value). *	1	6
Rubel et al, 2020 (50)	No description of the sampling strategy	0	Not justified.	0	No description of the response rate or the characteristics of the responders and the non-responders	0	Validated measurement tool. **	2	The study controls for the most important factor. *	1	Record linkage. **	2	The statistical test used to analyze the data is clearly described and appropriate, and the measurement of the association is presented, including confidence intervals and the probability level (p value). *	1	6

Hansen et al, 2019 (45)	No description of the sampling strategy	0	Not justified.	0	No description of the response rate or the characteristics of the responders and the non-responders	0	Validated measurement tool. **	2	The study controls for the most important factor. *	1	Record linkage. **	2	The statistical test used to analyze the data is clearly described and appropriate, and the measurement of the association is presented, including confidence intervals and the probability level (p value). *	1	6
Chen et al, 2021 (55)	No description of the sampling strategy	0	Not justified.	0	No description of the response rate or the characteristics of the responders and the non-responders	0	Validated measurement tool. **	2	The study controls for the most important factor. *	0	Independent blind assessment. **	2	The statistical test used to analyze the data is clearly described and appropriate, and the measurement of the association is presented, including confidence intervals and the probability level (p value). *	1	5
Dugas et al, 2018 (42)	No description of the sampling strategy	0	Not justified.	0	No description of the response rate or the characteristics of the responders and the non-responders	0	Validated measurement tool. **	2	The study controls for the most important factor. *	1	Record linkage. **	2	The statistical test used to analyze the data is clearly described and appropriate, and the measurement of the association is presented, including confidence intervals and the probability level (p value). *	1	6
Ellis et al, 2013 (43)	No description of the sampling strategy	0	Not justified.	0	No description of the response rate or the characteristics of the responders and the non-responders	0	Validated measurement tool. **	2	The study controls for the most important factor. *	1	Record linkage. **	2	The statistical test used to analyze the data is clearly described and appropriate, and the measurement of the association is presented, including confidence intervals and the probability level (p value). *	1	6
Tang et al, 2019 (53)	No description of the sampling strategy	0	Not justified.	0	No description of the response rate or the characteristics of the responders and the non-responders	0	Validated measurement tool. **	2	The study controls for the most important factor. *	1	Record linkage. **	2	The statistical test used to analyze the data is clearly described and appropriate, and the measurement of the association is presented, including confidence intervals and the probability level (p value). *	1	6
Rosa et al,2018 (54)	Truly representative of the average in the target population. * (all subjects or	1	Not justified.	0	No description of the response rate or the characteristics of the responders and the non-responders	0	validated measurement tool. **	2	The study controls for the most important factor. *	1	Record linkage. **	2	The statistical test used to analyze the data is clearly described and appropriate, and the measurement of the association is presented, including confidence intervals and the probability level (p value). *	1	7
Even et al,2021 (29)	No description of the sampling strategy	0	Not justified.	0	No description of the response rate or the characteristics of the responders and the non-responders	0	Validated measurement tool. **	2	The study controls for the most important factor. *	1	Record linkage. **	2	The statistical test used to analyze the data is clearly described and appropriate, and the measurement of the association is presented, including confidence intervals and the probability level (p value). *	1	6

Lokmer et al,2020 (30)	No description of the sampling strategy	0	Not justified.	0	No description of the response rate or the characteristics of the responders and the non-responders	0	Validated measurement tool. **	2	The study controls for the most important factor. *	1	Independent blind assessment. **	2	The statistical test used to analyze the data is clearly described and appropriate, and the measurement of the association is presented, including confidence intervals and the probability level (p value). *	1	6
Parbie et al, 2021 (49)	No description of the sampling strategy	0	Not justified.	0	No description of the response rate or the characteristics of the responders and the non-responders	0	Validated measurement tool. **	2	The study control for any additional factor. *	1	Record linkage. **	2	The statistical test used to analyze the data is clearly described and appropriate, and the measurement of the association is presented, including confidence intervals and the probability level (p value). *	1	6
Katsidzira et al, 2019 (46)	Truly representative of the average in the target population. * (all subjects or random...	1	Justified and satisfactory. *	1	No description of the response rate or the characteristics of the responders and the non-responders	0	Validated measurement tool. **	2	The study controls for the most important factor. *	1	Record linkage. **	2	The statistical test used to analyze the data is clearly described and appropriate, and the measurement of the association is presented, including confidence intervals and the probability level (p value). *	1	8
Ocvirk et al, 2020 (47)	No description of the sampling strategy	0	Not justified.	0	No description of the response rate or the characteristics of the responders and the non-responders	0	Validated measurement tool. **	2	The study controls for the most important factor. *	1	Record linkage. **	2	The statistical test used to analyze the data is clearly described and appropriate, and the measurement of the association is presented, including confidence intervals and the probability level (p value). *	1	6
Oduaran et al, 2020 (48)	No description of the sampling strategy	0	Not justified.	0	No description of the response rate or the characteristics of the responders and the non-responders	0	Validated measurement tool. **	2	The study controls for the most important factor. *	1	Record linkage. **	2	The statistical test used to analyze the data is clearly described and appropriate, and the measurement of the association is presented, including confidence intervals and the probability level (p value). *	1	6
Ayeni et al, 2018 (41)	Selected group of users	0	Not justified.	0	No description of the response rate or the characteristics of the responders and the non-responders	0	Validated measurement tool. **	2	The study controls for the most important factor. *	0	Independent blind assessment. **	2	The statistical test used to analyze the data is clearly described and appropriate, and the measurement of the association is presented, including confidence intervals and the probability level (p value). *	1	5
Lebba et al, 2016 (57)	No description of the sampling strategy	0	Not justified.	0	No description of the response rate or the characteristics of the responders and the non-responders	0	Validated measurement tool. **	2	The study controls for the additional factors. *	1	Record linkage. **	2	The statistical test used to analyze the data is clearly described and appropriate, and the measurement of the association is presented, including confidence intervals and the probability level (p value). *	1	6

Afolayan et al, 2019 (40)	No description of the sampling strategy	0	Not justified.	0	No description of the response rate or the characteristics of the responders and the non-responders	0	Validated measurement tool. **	2	The study controls for the most important factor. *	1	Independent blind assessment. **	2	The statistical test used to analyze the data is clearly described and appropriate, and the measurement of the association is presented, including confidence intervals and the probability level (p value). *	1	6
Afolayan et al, 2020 (39)	No description of the sampling strategy	0	Not justified.	0	No description of the response rate or the characteristics of the responders and the non-responders	0	Validated measurement tool. **	2	The study controls for the most important factor. *	1	Independent blind assessment. **	2	The statistical test used to analyze the data is clearly described and appropriate, and the measurement of the association is presented, including confidence intervals and the probability level (p value). *	1	6
Doumatey et al, 2020* (20)	Case definition is adequate with independent	1	consecuti ve or obviously represent	1	community controls selected, no history of disease (endpoint), same rate for both groups *	2	secure record (eg laboratory diagnosis) *	1	The study controls for the most important factor. *	1	secure record (eg laboratory diagnosis) *	1	The statistical test used to analyze the data is clearly described and appropriate, and the measurement of the association is presented,	1	8

Total score
1. Good quality: All criteria met (i.e. low for each domain) 2. Fair quality: One criterion not met (i.e. high risk of bias for one domain) or two criteria unclear 3. Poor quality: One criterion not met (i.e. high risk of bias for one domain) or two criteria unclear 4. Poor quality: Two or more criteria listed as high or unclear risk of bias

* - case control study

Appendix 3: An overview of the reported diets and their differences across the urbanization gradient

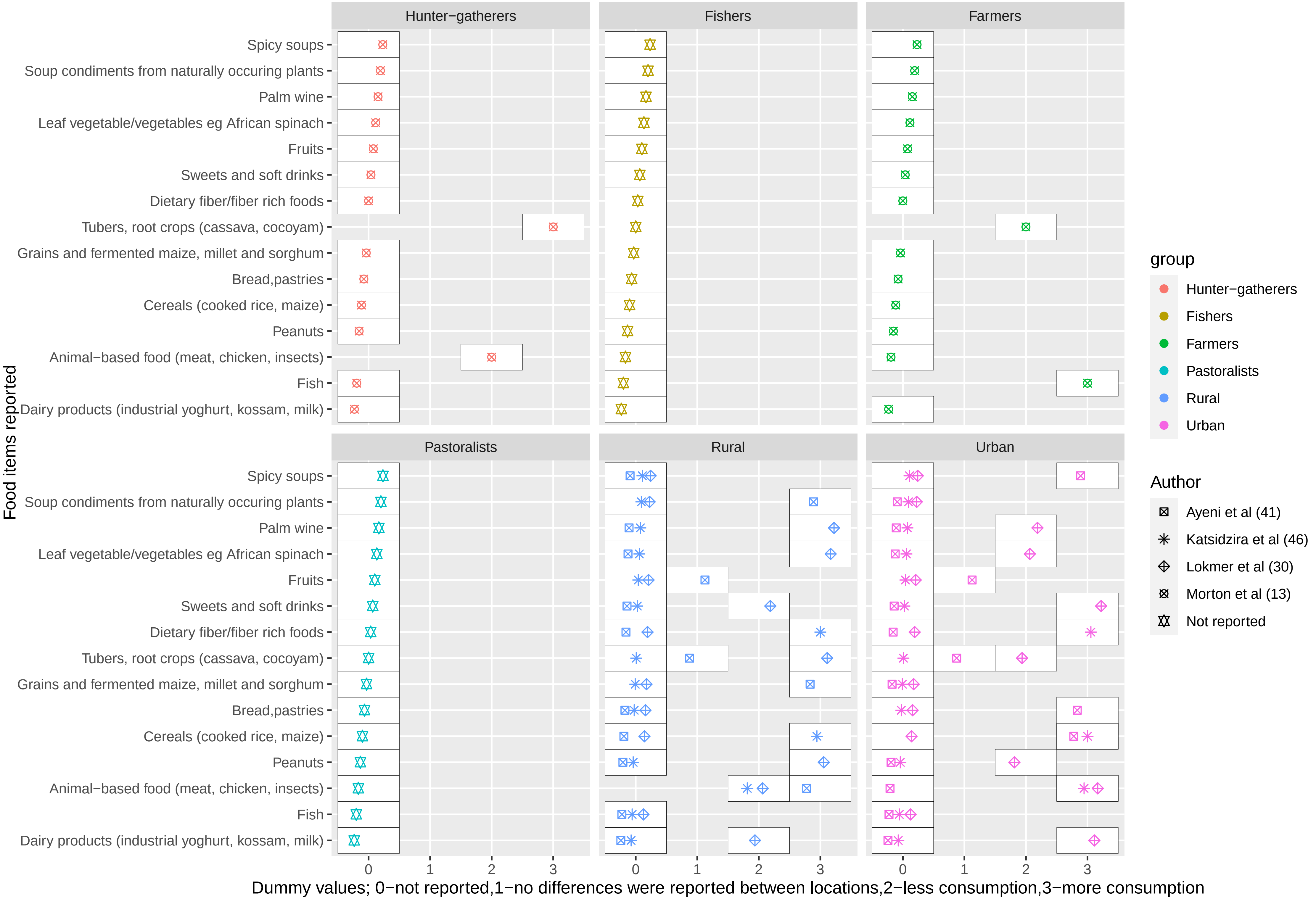

Appendix 4: An overview of the top 30 KEGG pathways reported in 5 studies included in the review

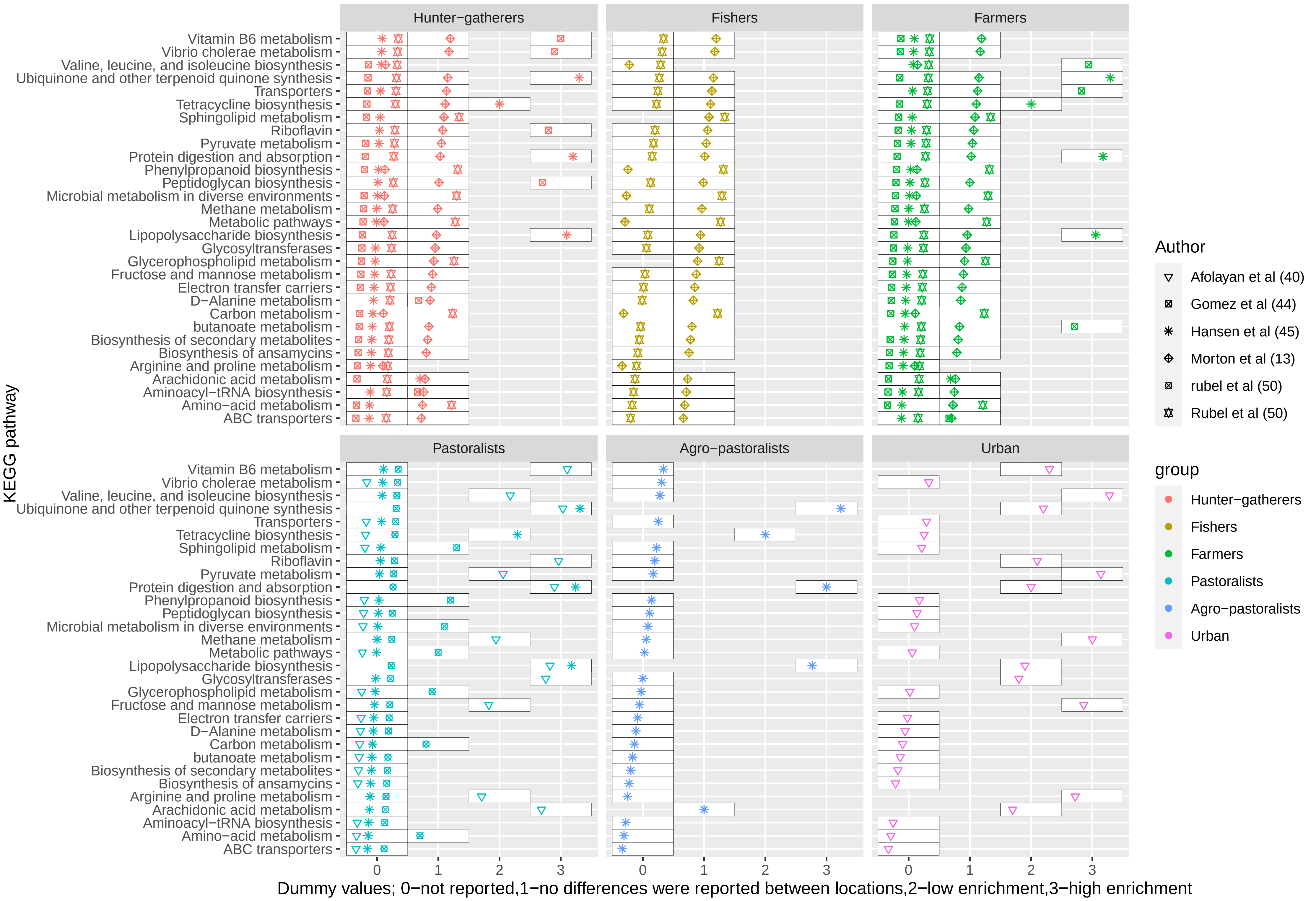

Appendix 5

A summary description of the methods used in the included studies

Statistical Analysis

Parametric and non-parametric tests were used to assess the differences in the gut microbiota diversity indices between groups and the association with variables of interest. Non-parametric tests that were used to assess the differences between taxa included Mann-Whitney U-test, Wilcoxon rank-sum test and Kruskal-Walli's test. Differences between groups were tested using the Analysis of variance (ANOVA) or permutation analysis of variance (PERMANOVA). T-tests were used for normally distributed data and the chi-squared test for categorical data. The correlation was tested using Spearman rho and Kendall- Tau correlation coefficients. P-values were widely used to test the level of significance of the differences in the composition of the gut microbiota while q-values were used to assess false discovery rates in two studies. One study assessed clustering using the Jensen-Shannon distance and two studies performed Principal component analysis using QIIME.

Intestinal parasites

The relationship between gut microbiota and intestinal parasites were assessed in seven studies as shown in the table below. The presence of intestinal parasites was assessed using microscopy and molecular techniques. Three studies identified intestinal parasites using microscopy (Lokmer, Morton and Chen), two used quantitative PCR and microscopy (Rose and Rubel) and two used qPCR only (Lebba and Even). Currently microscopy is still used as a gold standard in identifying intestinal parasites although its sensitivity is low. Molecular techniques which are better in identifying intestinal parasites are yet to cover all parasites.

Table 3: An overview of studies that assessed intestinal parasites across the urbanization gradient and techniques used

Author	Subsistence gradient	Intestinal parasites	Assessment
Gaei et al, 2021	Rural; semi-urban; urban	Relationship between gut microbiota and gut protozoa (blastocysts) and Entamoeba	mapping metagenomic shotgun data to respective genomes or 18S rRNA gene sequences
Lokmer et al, 2020	Rural; semi-urban; urban	Gut microbiota is partly shaped by the Entamoeba sp	microscopy
Morton et al, 2015	Paleolithic/ Hunter-gatherers; Rural; Agropastoral/subsistence farmers	Entamoeba sp is highly associated with the gut microbiota	microscopy
Rosa et al, 2018	Rural; semi-urban	Parasites were cleared and did not fully recover after 2 years to the original composition	quantitative real-time PCR (qPCR) and microscopy based Kato Katz smear
Rubel et al, 2020	Paleolithic/ Hunter-gatherers; Rural; Agropastoral/subsistence farmers	high number of parasite detections is correlated with a higher within-individual bacterial diversity	quantitative real-time PCR (qPCR) and microscopy based Kato Katz smear
Chen et al, 2021	Rural	there was a significant difference in alpha and beta diversity of helminth positive and helminth-negative study participants	microscopy
Lebba et al, 2016	Rural; urban	intestinal parasites shape the composition of the gut microbiota. Three species were identified and studied; Giardia duodenalis, Entamoeba spp and Blastocystis Hominis	Molecular identification using qPCR
